# Construction and Properties of SPI/PLA-PCL Composite Coating on Pure Titanium Surface

**DOI:** 10.3390/mi17080910

**Published:** 2026-07-29

**Authors:** Chunmei Wang, Congyi Zhu, Qi Zhang, Shuangsheng Zhang, Ling Zhang, Jiang Wu, Guoliang Zhang

**Affiliations:** School of Stomatology, Jiamusi University, Jiamusi 154000, China

**Keywords:** SPI/PLA–PCL composite coating, surface modification, micro-arc oxidation, spin coating, physical and chemical properties, biocompatibility

## Abstract

Titanium and titanium alloys have been widely used in clinical implants such as dental implants due to their high strength and corrosion resistance. However, their inherent biological inertness and mismatch with the elastic modulus of human bone tissue restrict bone healing and reconstruction. In this study, pure titanium was first modified by micro-arc oxidation (MAO), and then a biodegradable soybean protein isolate (SPI)/polylactic acid (PLA)–polycaprolactone (PCL) composite coating was prepared by using the spin-coating method. The coating was systematically characterized by scanning electron microscopy (SEM), an energy spectrometer (EDS), Fourier transform infrared spectroscopy (FT-IR), X-ray diffraction (XRD), and atomic force microscopy (AFM), confirming that the composite coating was successfully prepared. An evaluation of the physical and chemical properties showed that the introduction of SPI significantly improved the hydrophilicity, surface roughness and adhesion to the substrate of the coating, regulated the degradation rate, and reduced the elastic modulus to the range of 10–30 GPa, which matches human bone tissue. At the same time, it enhanced corrosion resistance. In vitro MC3T3-E1 osteoblast experiments showed that the coating containing 50% SPI showed better cell compatibility, adhesion ability and osteogenic inducibility, and all groups had good blood compatibility. The SPI/PLA–PCL composite coating effectively improves the biological activity of the pure titanium surface and provides a feasible strategy for the surface modification of titanium implants and bone defect repair.

## 1. Introduction

In the treatment of bone defects caused by congenital malformation, inflammation, tumors, and trauma, the application of medical implants is becoming more and more extensive [1]. Titanium and titanium alloys have been widely used in clinics as dental implants, artificial hip joints, bone nails and other medical implants because of their advantages of low density, good biocompatibility and basically no harm to the human body [2]. However, there are some shortcomings in the application of titanium and titanium alloys, such as the influence of biological inertia [3,4] and the great difference in elastic modulus between titanium and titanium alloys and normal human bone tissues [5,6]. Scholars try to improve the physical and chemical properties of the implant surface, promote cell proliferation and growth, accelerate bone union, etc., through surface treatment technologies for titanium and titanium alloys, in order to obtain better clinical effects [7,8].

Micro-arc oxidation is a method for the surface treatment of titanium and titanium alloys [9,10]. The advantages of micro-arc oxidation include the following: (1) the ceramic membrane formed is closely and evenly bonded with the titanium matrix; (2)the surface of the micro-arc oxidation coating is porous, and the pore size ranges from several microns to several tens of microns, which provides adhesion space for the combination of other bioactive factors and drug-loaded microspheres [11,12,13,14]. Therefore, this topic plans to modify the surface of the titanium substrate by micro-arc oxidation to form a porous structure on its surface, so as to enhance the bonding force between SPI/PLA–PCL organic materials and titanium substrate.

Polylactic acid (PLA) has good biocompatibility, biodegradability, and plasticity, and is widely used in biomedical fields as a material for medical surgical sutures, drug sustained-release carriers, and internal fixation devices for fractures [15,16,17]. In order to overcome the brittleness of PLA materials, it is necessary to modify PLA raw materials [18]. Polycaprolactone (PCL) as a medical material has good biocompatibility, biodegradability, moderate mechanical strength and high flexibility [19,20]. The modification of PLA by blending PLA with PCL can change the modulus and tensile strength of the blend while maintaining biodegradability [21,22].

Soy protein isolate (SPI) has good cell compatibility, osteoinductivity, hydrophilicity, and biodegradability, and is suitable for food packaging, plastic, medical sutures, dressings, fibers, biofilms, and other applications [23,24,25]. However, soy protein isolate has the characteristics of poor water resistance, poor mechanical properties and easy degradation [26].

In the early stage, the research team took advantage of the complementary properties of composite materials to toughen and modify PLA with PCL, enabling PLA to overcome its high brittleness. By doping with SPI, the resulting SPI/PLA–PCL composite overcomes the problems of poor mechanical properties of SPI, poor hydrophilicity of PLA, and slow biodegradation [27]. The synthesis of the SPI/PLA–PCL composite was completed. It was initially found that the doping of SPI can improve the osteoinduction regeneration of the PLA–PCL composite. The purpose of this research is to use micro-arc oxidation technology to treat the titanium surface and improve its biological inertness. A porous and dense structure is formed on its surface as a “bridge” to form a titanium surface composite coating. The SPI/PLA–PCL composite organic material is then spin-coated on the titanium surface after micro-arc oxidation treatment. Through the characterization of the composite coating, physical and chemical properties, biological properties (blood compatibility, biocompatibility, osteogenic inducibility) and other related tests, the feasibility of improving the physical, chemical and biological properties of the titanium-based surface is explored.

## 2. Materials and Methods

### 2.1. Experimental Reagents and Instruments

#### 2.1.1. Experimental Reagents

The detailed information of the experimental reagents is summarized in Table 1.

#### 2.1.2. Experimental Instruments

Detailed information on the experimental instruments is summarized in Table 2.

### 2.2. Experimental Method

#### 2.2.1. Preparation of Titanium Parts

TA2 pure titanium plates were selected and cut into circular disks with a diameter of 15 mm and a thickness of 2 mm via laser cutting. The specimens were sequentially ground with water-resistant silicon carbide (SiC) abrasive papers of 400#, 800# and 1200# grit. Following grinding, the titanium substrates were ultrasonically cleaned sequentially in acetone, anhydrous ethanol and deionized water for 15 min each, then dried in a constant-temperature oven for subsequent use.

#### 2.2.2. Micro-Arc Oxidation Treatment of Titanium Sheets

The titanium sheets were subjected to micro-arc oxidation (MAO) treatment for 5 min using the electrolyte system reported in reference [28], which consisted of 15 g/L trisodium phosphate (Na_3_PO_4_), 5 g/L sodium silicate (Na_2_SiO_3_), 5 g/L potassium hydroxide (KOH), and 6 g/L potassium fluoride (KF). The electrical parameters were set as follows: pulse frequency of 500 Hz, pulse width of 100 μs, and duty cycle of 5%. After MAO treatment, the specimens were thoroughly rinsed with deionized water, dried, and stored for subsequent experiments.

#### 2.2.3. Preparation Process of SPI-PLA/PCL Composite Coating on Pure Titanium by MAO

A total of 0.5 g of the PLA–PCL blend (PLA:PCL mass ratio = 85:15) and SPI powders at various mass fractions (0%, 30%, 50%) were weighed using an electronic analytical balance. The raw materials were dissolved in 20 mL dichloromethane and magnetically stirred at 400 rpm until complete dissolution.

MAO-modified titanium substrates were fixed on a spin coater. A volume of 0.2 mL of the prepared solution was dropped onto the titanium surface with a syringe, followed by spin coating at 3000 r/min for 20 s [29]. After the spin coater stopped working, the samples were taken out. Four experimental groups and one control group were set up: Ti-0% SPI/PLA–PCL, Ti-30% SPI/PLA–PCL, Ti-50% SPI/PLA–PCL, MAO-only group, and smooth pure Ti served as the blank control group. All samples were freeze-dried and sterilized for subsequent experiments.

### 2.3. Characterization Analysis

#### 2.3.1. Field-Emission Scanning Electron Microscopy–Energy Spectroscopic Analysis (SEM-EDS)

The surface morphology of the samples was observed using a field-emission scanning electron microscope. Samples were sprayed with gold before testing to improve electrical conductivity. The composition and distribution of elements on the coating surface were analyzed using an energy spectrometer.

#### 2.3.2. X-Ray Diffraction (XRD)

The crystal structure of the sample was characterized using an X-ray diffractometer with Cu-Kα radiation, a working voltage of 40 kV, a current of 30 mA, a scanning range of 2θ = 10°–80°, and a scanning speed of 2°/min.

#### 2.3.3. Fourier Transform Infrared Spectroscopy (FT-IR)

Coating powders were scraped from the sample surfaces. Approximately 1 mg of the powder sample was uniformly mixed and ground with 500 mg of dried potassium bromide, and subsequently pressed into a 13 mm diameter pellet [30]. FT-IR spectra were collected in the wavenumber range of 4000–400 cm^−1^ with a resolution of 4 cm^−1^.

#### 2.3.4. Water Contact Angle Test

The static water contact angle of sample surfaces was determined by a contact angle goniometer. The volume of the distilled water droplet was 1 μL, and each group of specimens was subjected to three parallel measurements.

#### 2.3.5. Atomic Force Microscopy (AFM)

AFM acquires high-resolution topographical and physical property information about a sample by detecting interatomic forces between the probe and the sample surface. In this study, roughness measurements were performed on dried specimens, with a test area of 10 μm × 10 μm selected.

### 2.4. Physical and Chemical Performance Tests

#### 2.4.1. In Vitro Degradation Experiment and pH Determination

In accordance with ISO 10993-15:2019 Qualitative and Quantitative Analysis of Degradation Products of Metals and Alloys [31], 50 mL plastic centrifuge tubes were prepared. Four groups of coated titanium specimens were weighed (initial weight, W_0_) and placed into the centrifuge tubes, followed by the addition of 30 mL of phosphate-buffered saline (PBS). The tubes were incubated in a constant-temperature incubator at 37 °C, and the pH value of the immersion solution was monitored regularly. Specimens were harvested at predetermined time points: days 1, 3, 5, 7, and thereafter weekly up to 12 weeks. After removal, the samples were rinsed three times with deionized water, dried, weighed (W_t_), and their data were recorded.

#### 2.4.2. In Vitro Mineralization Experiment

Specimens were immersed in simulated body fluid (SBF), and the SBF solution was renewed every two days. Samples were collected on days 7 and 14. After drying, scanning electron microscopy (SEM) was used to observe the surface mineralized morphology.

#### 2.4.3. Elastic Modulus Test

The surface elastic modulus of the samples was tested using a nano-indenter, and 3 samples were tested in each group. The mechanical behavior of the composite coatings was evaluated using a nano-indenter. Load versus penetration depth curves were recorded under controlled constant conditions in the dynamic loading range—maximum load: 5.00 mN; loading rate: 10.00 mN/min; unloading rate: 10.00 mN/min; and pause: 5.0 s.

#### 2.4.4. Coating Bond Strength Test

Coating adhesion strength was evaluated using a scratch tester. The applied load was 30 N, the loading rate was 30 N/min, and the scratch length was 3 mm. Three random scratches were made on each specimen, and the average value was calculated.

#### 2.4.5. Corrosion Resistance Test

Potentiodynamic polarization measurements were performed in SBF using an electrochemical workstation equipped with a standard three-electrode system. The specimen served as the working electrode, an Ag/AgCl electrode acted as the reference electrode, and a platinum plate was used as the counter electrode. Three parallel tests were conducted for each group, and Origin software was employed for data analysis.

### 2.5. Biological Evaluation

#### 2.5.1. Hemolysis Test

Sterilized specimens were immersed in 0.9% normal saline. Fresh rabbit blood was anticoagulated with EDTA and then diluted with normal saline at a ratio of 1:1.25. A negative control group (normal saline), a positive control group (triple-distilled water) and experimental groups (*n* = 3) were established. After pre-incubation at 37 °C, diluted rabbit blood was added and incubated for 1 h. Following centrifugation, the supernatant was collected, and the absorbance at 545 nm was detected. The hemolysis rate was calculated according to the given formula.

#### 2.5.2. Cell Experiments

Mouse pre-osteoblast MC3T3-E1 cells were cultured in α-MEM medium supplemented with 10% fetal bovine serum and 1% penicillin–streptomycin solution. Cells were incubated at 37 °C under a 5% CO_2_ atmosphere for routine subculture, cryopreservation, and resuscitation.

#### 2.5.3. Cell Proliferation (CCK-8)

Sterilized specimens were placed in 24-well plates, and cells were seeded at a density of 3.0 × 10^4^ cells/well. CCK-8 solution was added on days 1, 3 and 5 of incubation. After 1 h of incubation, the absorbance at 450 nm was determined.

#### 2.5.4. Cytotoxicity (CCK-8)

The cell seeding and culture procedures were identical to those described above. Cell viability (%) was calculated using the following formula:

Cell viability (%) = [(OD experimental group − OD blank group)/(OD negative control group − OD blank group)] × 100%

#### 2.5.5. Laser Confocal Microscopy Observations

Cells were seeded at 2 × 10^4^ cells/well and cultured for 48 h and 72 h. After fixation and permeabilization, cells were stained with phalloidin and DAPI. Cell morphology was observed under a laser scanning confocal microscope.

#### 2.5.6. Alizarin Red Staining

After 7 and 14 days of osteogenic induction culture, the cells were fixed and stained with a 2% alizarin red solution. Calcium nodules were observed under an optical microscope. Subsequently, 10% cetylpyridinium chloride solution was used for dissolution, and absorbance at 562 nm was measured for semi-quantitative analysis.

#### 2.5.7. Alkaline Phosphatase (ALP) Activity

Cells were cultured in osteogenic induction medium supplemented with specimen extracts for 7 and 14 days. After cell lysis, ALP activity was detected at 405 nm using a commercial kit. Total protein content was determined at 520 nm via the BCA method for normalization.

### 2.6. Statistical Analysis

Measurement data are expressed as mean ± standard deviation (Mean ± SD). Using SPSS 22.0 software, data comparison between two groups was analyzed by *t* test, and comparisons among multiple groups were analyzed by one-way ANOVA. *p* < 0.05 was considered to be statistically different. Drawings were prepared using Origin2021 and GraphpadPrism9.5.

## 3. Results

### 3.1. Characterization of Each Group of Specimens

#### 3.1.1. SEM of Each Group of Specimens

**Figure 1:**
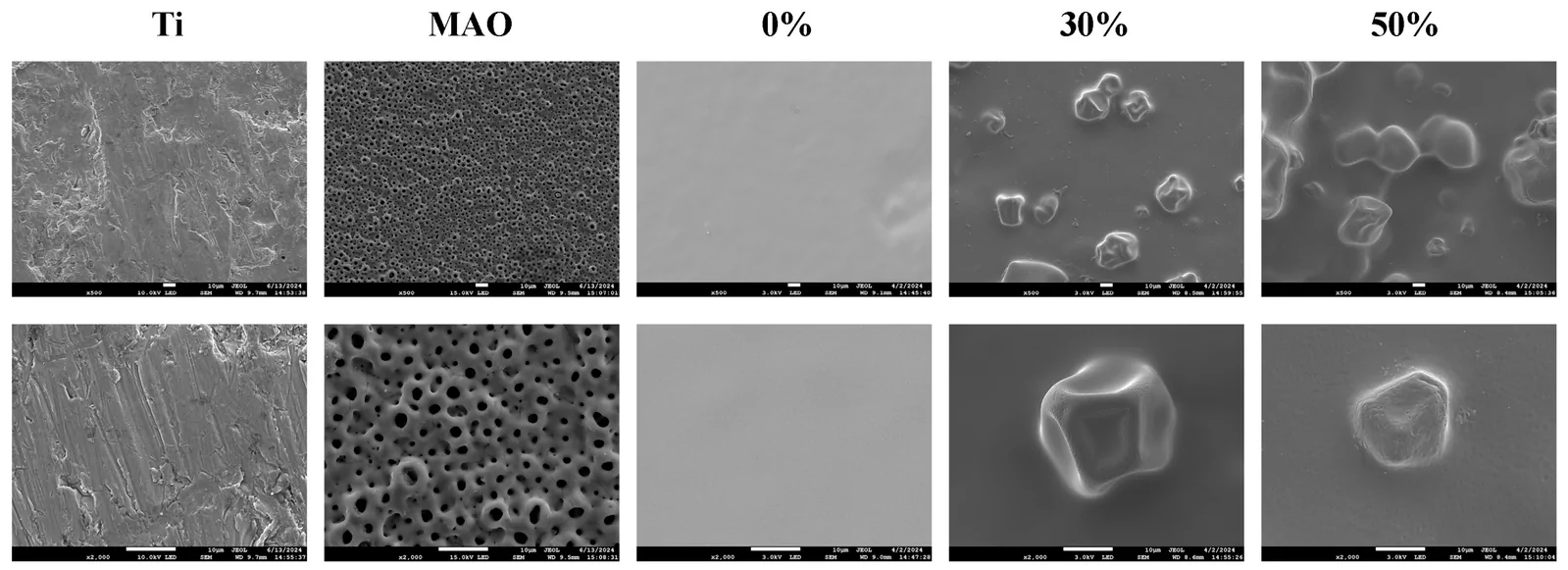
Scanning electron microscope images of each group of specimens.

The surface morphologies of all groups are presented in Figure 1. Scratches derived from sandpaper grinding can be observed on the bare pure Ti substrate. After MAO treatment, the pure titanium surface exhibited a porous structure with pore diameters ranging from 1 to 10 μm and relatively uniform pore distribution. The surface displayed a crater-like morphology, which greatly enlarged the specific surface area, accompanied by minor microcracks between adjacent pores. In the 0% SPI group, a smooth PLA–PCL coating was uniformly deposited on the MAO surface, completely covering the original micropores and microcracks of the MAO layer. In the 30% SPI group, SPI particles with sizes of approximately 10–40 μm were dispersed within the smooth PLA–PCL matrix, and the composite coating was homogeneously distributed on the MAO substrate. In the 50% SPI group, the number of SPI particles was significantly higher than that in the 30% SPI group.

#### 3.1.2. EDS of Each Group of Specimens

**Figure 2:**
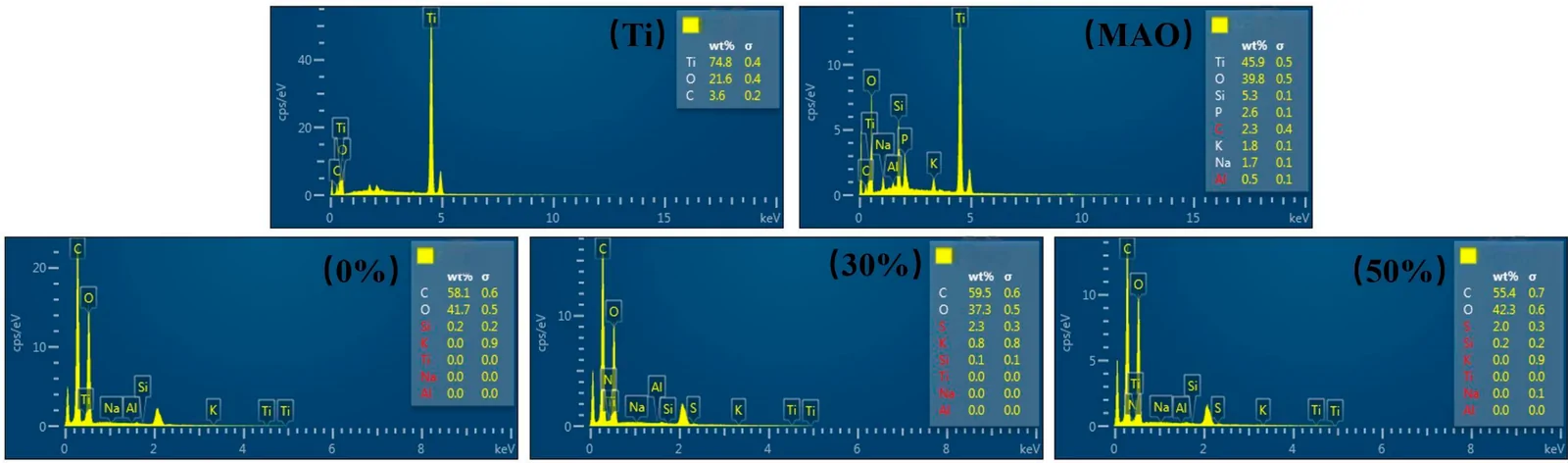
EDS patterns of each group of specimens.

The EDS of each group of samples in the experiment is shown in Figure 2: Smooth Ti group: The surface elements of the specimen are mainly Ti, O, and C, which are derived from the surface of pure titanium. MAO group: In addition to the three elements of Ti, O, and C, Si, P, K, Na and other elements from the electrolyte can also be seen, among which P and Si come from PO43- and SiO32- in the electrolyte, respectively. The 0% group: On the coating surface, the content of C and O elements increases. C and O mainly come from PLA and PCL, and elements such as Ti, P, Si, K, and Na are almost undetectable, indicating that the PLA–PCL coating has completely covered the MAO surface. The 30% and 50% groups: Compared with the 0% group, in addition to containing C and O elements, S elements are also detected. S comes from SPI, indicating that SPI is successfully loaded in the coating.

#### 3.1.3. FT-IR of nSPI/PLA-PCL Composite Coating

**Figure 3:**
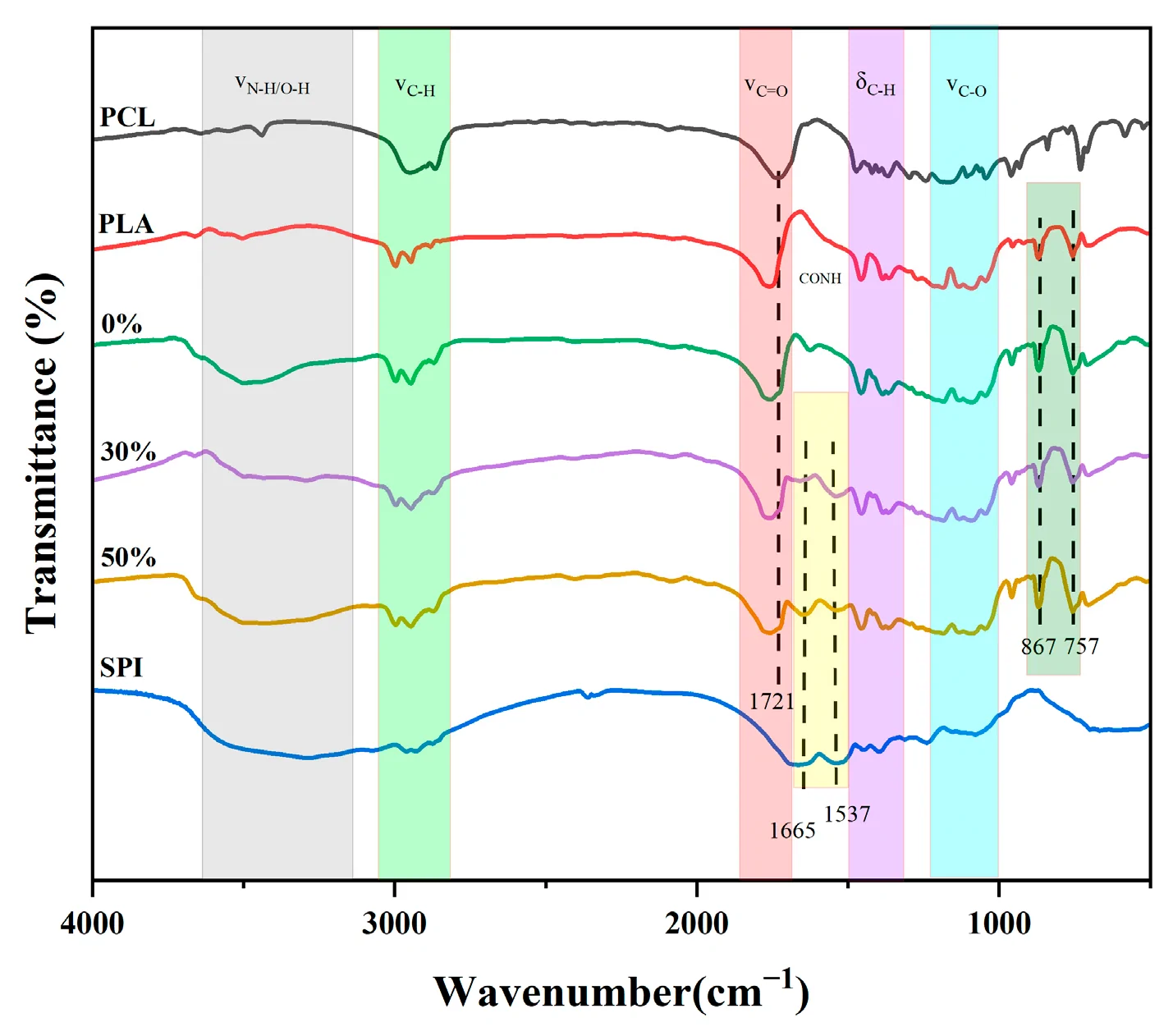
FT-IR diagram of NSPI/PLA–PCL composite coating.

By FT-IR (as shown in Figure 3), three groups of organic coating samples (0%, 30% and 50%) were identified through compound and functional group analysis. It was found that for PCL, the absorption peak at 3330 cm^−1^ was the stretching vibration of O-H, and the absorption peak at 1721 cm^−1^ was the stretching vibration of C=O; for PLA, the absorption peak at 3502 cm^−1^ is the stretching vibration of hydroxyl O-H, at 3000–2800 cm^−1^, it is the stretching vibration of alkyl C-H, and the absorption peaks at 876 and 757 cm^−1^ are related to the crystalline phase and amorphous phase of PLA. SPI: The absorption peaks at 1665 cm^−1^ and 1537 cm^−1^ are the stretching vibration of amide I band and amide II; 0% group: the absorption peaks are mainly similar to PLA, and the absorption peaks at 876 cm^−1^ and 757 cm^−1^ in the 0%, 30% and 50% groups are related to the crystalline phase and amorphous phase of PLA, which indicates the addition of PLA. There was a weak absorption peak at 1721 cm^−1^ in the 0% group, and there was this absorption peak at 30% and 50% content, which indicated the introduction of PCL. With the increase in SPI content, the absorption peaks at 1665 cm^−1^ and 1537 cm^−1^ in the 30% and 50% groups are the stretching vibrations of amide I band C=O and the bending vibration of amide II band C-N-H, which become more obvious with the increase in SPI content. These two absorption peaks do not appear in the 0% group, which indicates the introduction of SPI.

#### 3.1.4. XRD Patterns of Each Group of Specimens

**Figure 4:**
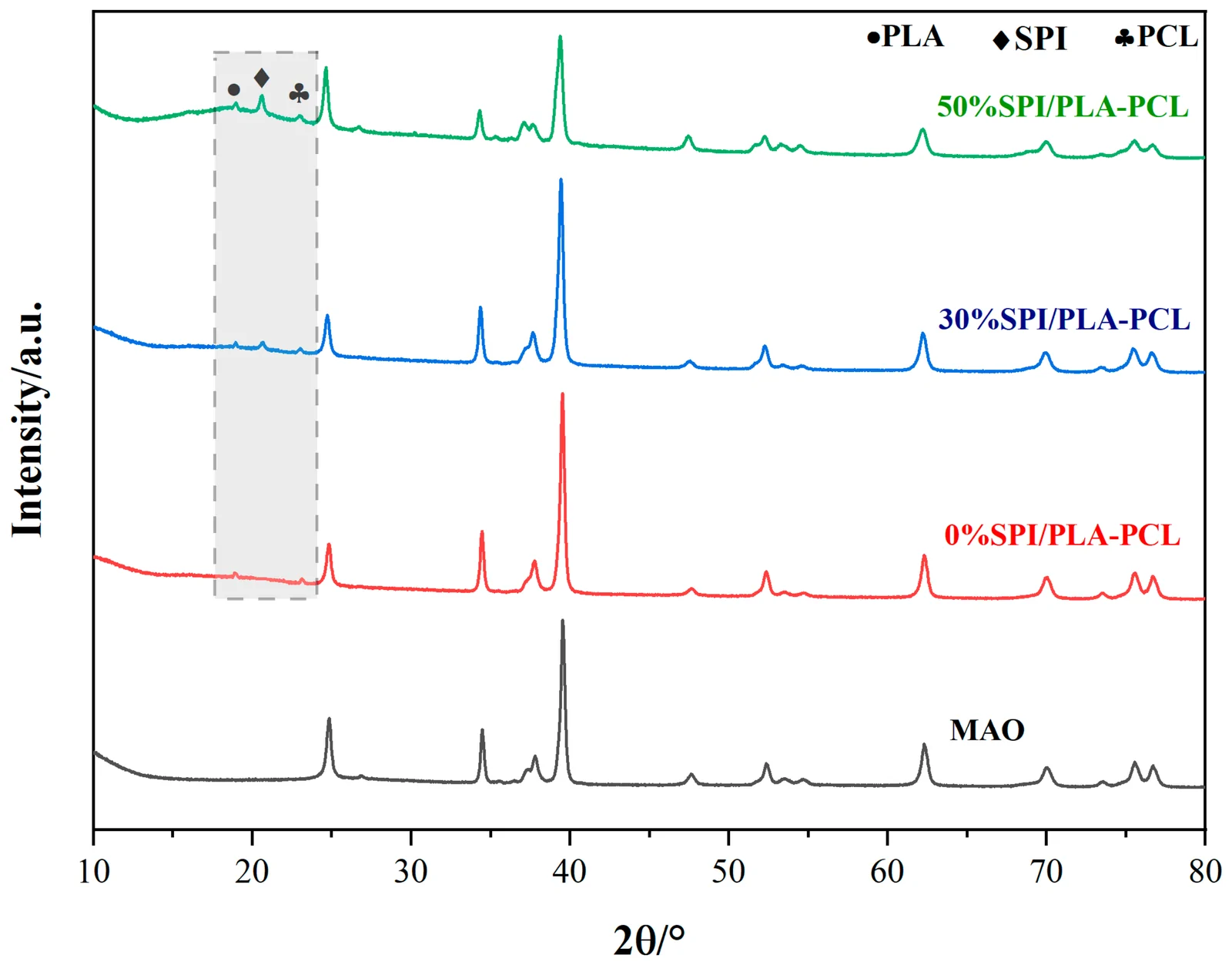
X-ray diffraction patterns of each group of specimens.

Figure 4 shows the XRD patterns of all coating groups and the MAO substrate. In the scanning range of 10–20°, all coated groups (0%, 30%, and 50% SPI) exhibited a weak diffraction peak at 2θ = 18.9°, which corresponded to the (203) crystal plane of PLA, matching the standard PDF No. 06-0405. A diffraction peak at 2θ = 24.2° was attributed to the crystalline structure of PCL, consistent with the standard PDF No. 49-2207. This peak presented low intensity due to the low PCL proportion in the blend, verifying the existence of PCL within the coating system. Characteristic diffraction peaks of SPI appeared at approximately 2θ = 21° in both the 30% and 50% SPI groups, which agreed with the standard PDF No. 62-1863, indicating successful SPI loading. With increasing SPI content, the SPI diffraction peaks of the 50% group displayed broader width and higher intensity compared with the 30% group. Compared with the pure MAO substrate, no additional impurity diffraction peaks were observed in any of the coated groups. These results demonstrate that the composite coatings were successfully fabricated without introducing crystalline impurities, which is consistent with the FT-IR analysis.

#### 3.1.5. Water Contact Angle of Each Group of Specimens

**Figure 5:**
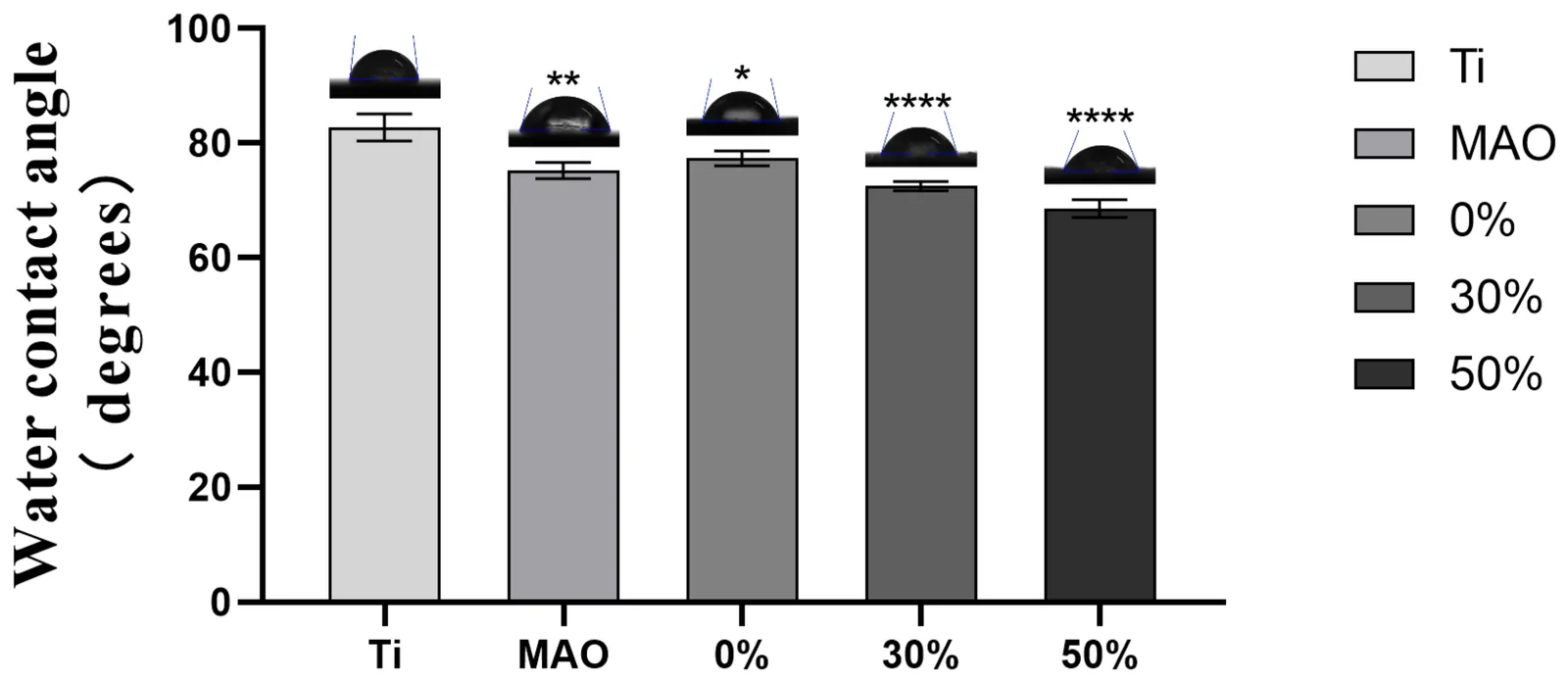
Water contact angle of each group of specimens. *, *p* < 0.05; **, *p* < 0.01; ****, *p* < 0.0001; *n* = 3.

The hydrophilicity of the material surface plays a key role in the interaction with cells [32]. The surface contact angle of each group of titanium parts is shown in Figure 5. The Ti group exhibited the largest water contact angle, reaching 82.76 ± 2.37°. After micro-arc oxidation treatment, the water contact angle of the titanium substrate decreased to 75.22 ± 1.38°. The contact angle of the 0% coating group was slightly higher than that of the MAO group, which was 77.34 ± 1.26°, owing to the intrinsic hydrophobic property of the PLA–PCL polymer. The water contact angles of the 30% and 50% groups were 72.51 ± 0.80° and 68.55 ± 1.55°, respectively. The incorporation of SPI reduced the hydrophobicity of the PLA–PCL composite coating, namely enhancing the surface hydrophilicity. Statistical analysis showed that the MAO, 0%, 30% and 50% groups had significantly lower water contact angles than the pristine Ti group (*p* < 0.05), demonstrating that MAO pretreatment and subsequent organic composite coating modification effectively optimized the surface wettability of titanium substrates.

#### 3.1.6. Surface Roughness of Each Group of Specimens

**Figure 6:**
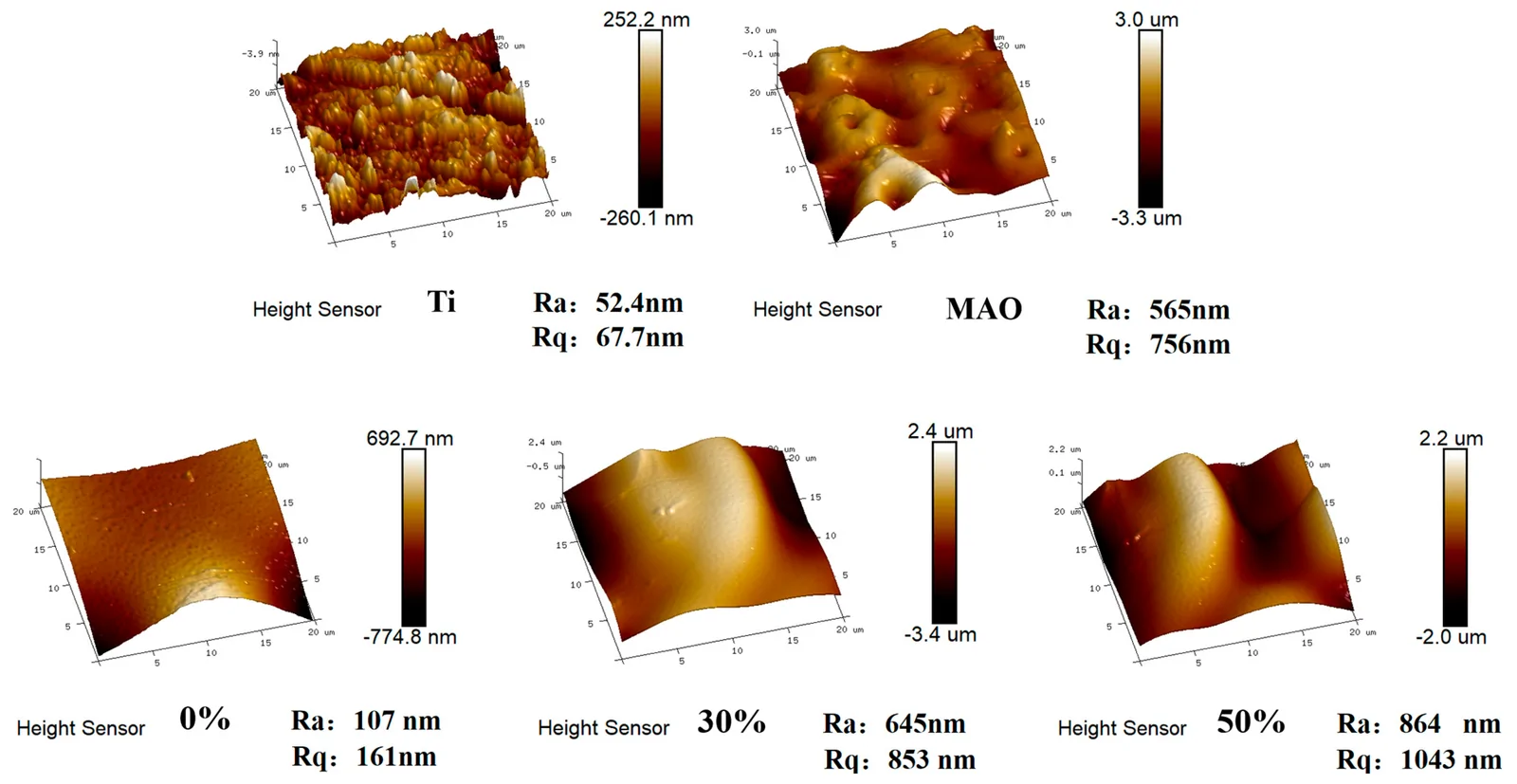
AFM images of the surfaces of each group of specimens.

Atomic force microscopy (AFM) was adopted to characterize the surface roughness of five groups of samples (Ti, MAO, 0% SPI, 30% SPI and 50% SPI), and representative full-view 3D topography images of each group are displayed in Figure 6. Ra refers to the arithmetic average roughness, which is calculated as the average absolute deviation of surface height relative to the reference plane; Rq denotes the root-mean-square roughness, derived from the root mean square of surface height fluctuations. Compared with Ra, Rq is more sensitive to sharp surface protrusions and pits, which can better reflect the overall undulation characteristics of coatings.

The surface roughness follows the order: Ti < 0% SPI < MAO < 30% SPI < 50% SPI. The pristine Ti substrate possesses a smooth surface with Ra = 52.4 nm and Rq = 67.7 nm. After micro-arc oxidation, abundant porous crater structures are formed on the Ti surface, which greatly increase roughness, with Ra and Rq of the MAO coating reaching 565 nm and 756 nm, respectively. The pure PLA–PCL coating (0% SPI) covers the MAO substrate and fills micro-pores, thus smoothing the surface and reducing roughness to Ra = 107 nm and Rq = 161 nm, which is still higher than bare Ti substrate. With the increase in SPI content, SPI components gradually enrich on coating surfaces and form regular microscale aggregated protrusions, leading to continuous growth of surface roughness. The 30% SPI group has values of Ra = 645 nm and Rq = 853 nm, while the 50% SPI group exhibits the maximum roughness of Ra = 864 nm and Rq = 1043 nm due to abundant surface aggregates. This gradient variation provides an effective strategy to regulate surface micro-topography, and these micro-protrusions can offer more binding sites to facilitate cell adhesion.

### 3.2. Physical and Chemical Properties of Each Group of Specimens

#### 3.2.1. Degradation Performance Test and pH Value Determination of Each Group of Specimens

**Figure 7:**
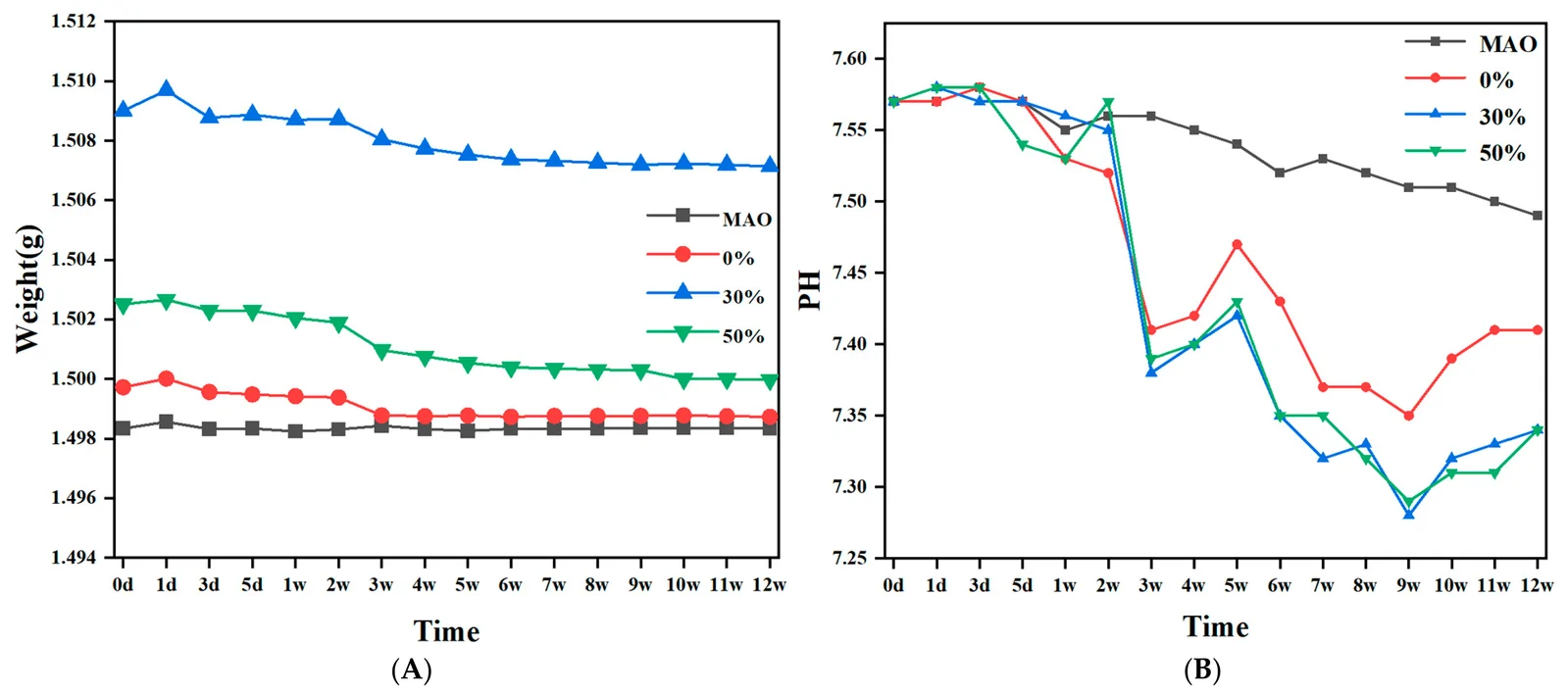
(**A**) Mass change curve of each group of specimens in PBS solution, and (**B**) pH value change in each group of specimens in PBS solution.

Figure 7A depicts the weight evolution of various coatings during 12 weeks of degradation in PBS solution. With prolonged immersion, all samples (MAO, 0%, 30%, and 50% groups) displayed an initial slight weight gain, followed by a continuous and gradual weight loss.

For the MAO group, temporary weight gain was observed from 0 to 1 day, which was attributed to the infiltration of PBS into the porous ceramic structure of MAO. After 1 day, slow surface dissolution and erosion of the MAO ceramic layer occurred. The mass loss from material dissolution exceeded the mass increment derived from liquid absorption, resulting in a slight weight reduction. From day 3 to 12 weeks, the MAO layer possessed excellent chemical stability with a low erosion rate, leading to minor mass fluctuations and an overall stable weight. The 0%, 30%, and 50% organic composite coatings shared an identical variation tendency. During 0–1 day, abundant PBS was absorbed into the interstitial spaces of polymer chains, while hydrolysis of the coatings was negligible. The mass increase caused by liquid adsorption outweighed the mass loss from degradation, leading to weight gain, and the 30% group achieved the maximum increment. After 1 day, continuous water permeation triggered hydrolysis of ester bonds in PLA–PCL, and small-molecular degradation products leached out constantly. The rate of mass loss from degradation surpassed that of liquid absorption, resulting in sustained weight reduction. The degradation rate increased markedly between 2 and 3 weeks, as PBS had fully penetrated the entire thickness of the organic coating, inducing extensive cleavage of polymer backbones and massive release of degradation products into the solution. From 3 to 12 weeks, most readily hydrolyzable components were exhausted, and the degradation rate of the residual structure slowed down, accompanied by gradual weight loss. Throughout the test, the weight loss of the 30% SPI group was greater than that of the 0% group, and the 50% group exhibited the most significant weight loss.

Figure 7B shows the pH variation curves of PBS solution for all specimens over the 12-week degradation period. The pH value of the MAO group declined slowly with negligible fluctuations, reaching approximately 7.49 at 12 weeks. All organic coating groups (0%, 30%, 50%) followed a consistent pH trend: slight decrease, rebound, a second drop to the minimum value, and subsequent slow recovery. At the early immersion stage, a small amount of lactic-acid-like acidic substances generated by PLA–PCL hydrolysis reduced the solution pH, which was then neutralized by the PBS buffer system, giving rise to pH recovery. During weeks 2–3, large-scale hydrolysis of the coatings released a large quantity of acidic degradation products rapidly, driving the pH to its lowest level. The pH reduction in the 30% and 50% groups was markedly more pronounced than that in the 0% group. Afterwards, the readily degradable components were consumed, the release of acidic substances diminished, and the buffering capacity of PBS became prominent, resulting in a gradual rise in pH. At the end of the 12-week immersion, the pH values of the MAO, 0%, 30%, and 50% groups were about 7.49, 7.41, 7.34 and 7.34, respectively. Overall, the pH of all groups remained above 7.25 throughout the experiment, maintaining a neutral to slightly alkaline environment. The PBS buffer system can effectively neutralize acidic substances produced by polyester degradation, avoiding excessively low local pH values. Therefore, inflammatory reactions in surrounding tissues caused by microenvironmental acidification can be theoretically avoided.

#### 3.2.2. Mineralization In Vitro

**Figure 8:**
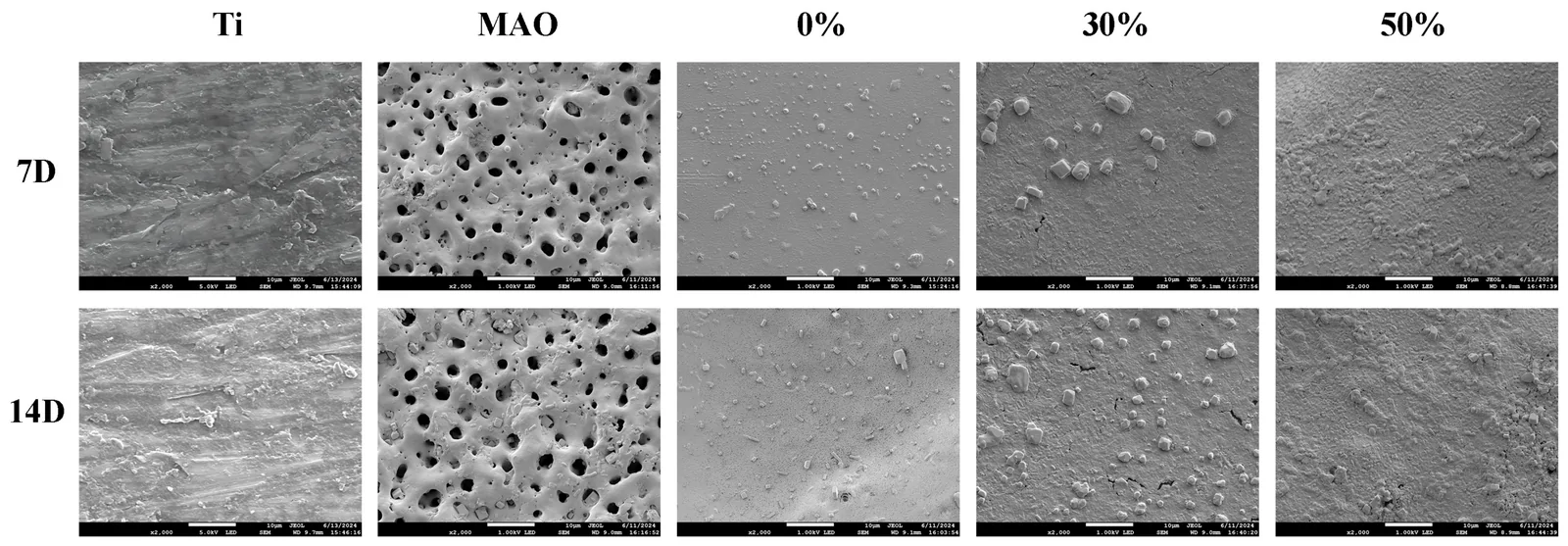
SEM images of each group of specimens soaked in SBF for 7 d and 14 d.

SEM micrographs of in vitro mineralization are displayed in Figure 8. After 7 and 14 days of immersion in simulated body fluid (SBF), short rod-shaped mineral crystals were formed on the surfaces of all experimental specimens. The pure Ti group exhibited the least amount of mineral deposition. In comparison, mineral crystal deposition gradually increased in the 0%, 30%, and 50% SPI groups with elevated SPI content. During SBF immersion, the composite coatings underwent simultaneous degradation and mineralization. Surface cracks, increased roughness, and thickened surface layers were observed, indicating uneven degradation behavior. Overall, both mineralization and degradation degrees were more pronounced at 14 days than at 7 days of immersion.

**Figure 9:**
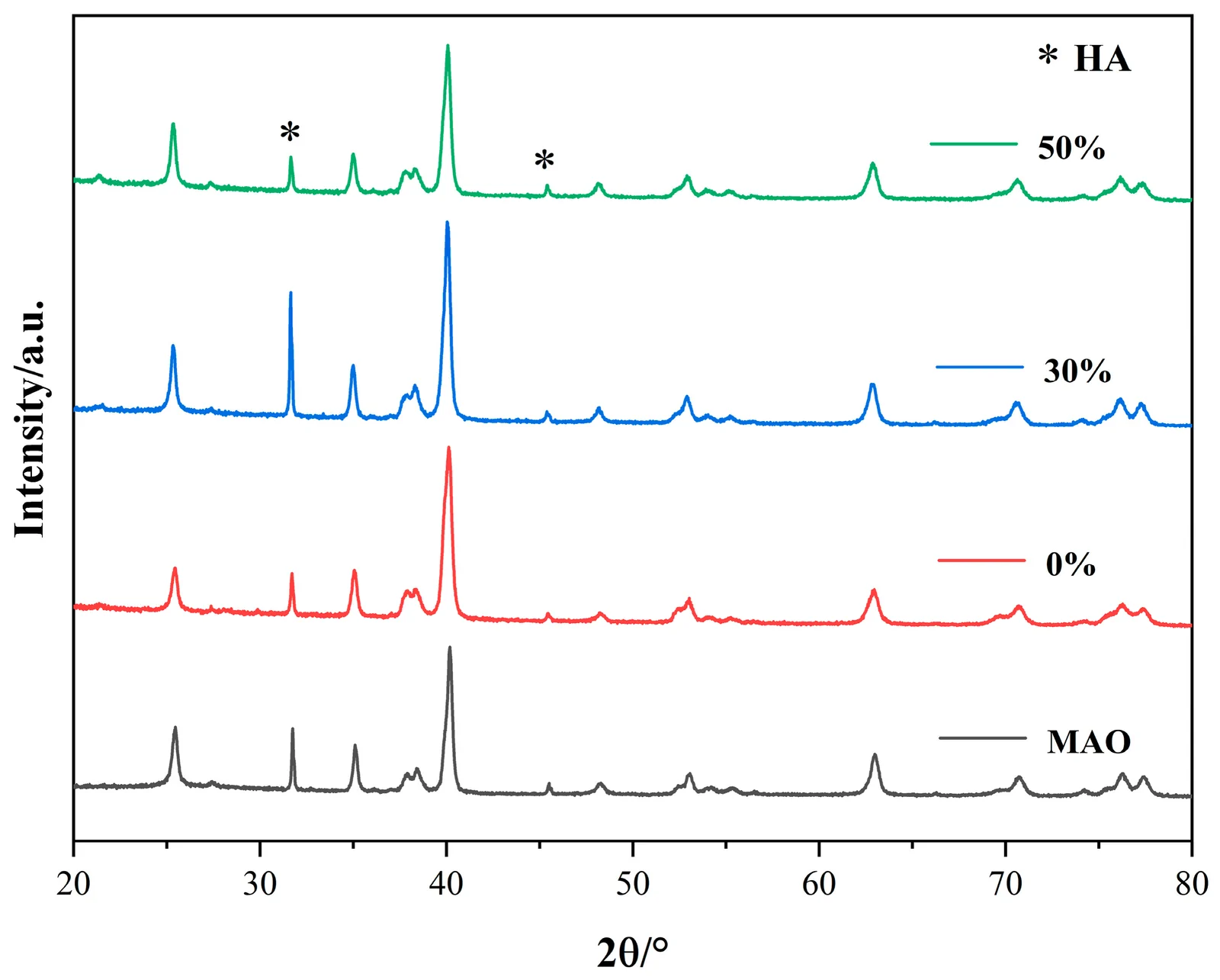
XRD patterns of each group of specimens soaked in SBF for 14 days.

As shown in Figure 9, after 14 days of immersion in simulated body fluid (SBF), the XRD spectra of all groups exhibited characteristic apatite diffraction peaks at 2θ = 31.77° (211) and 2θ = 45.8°, which matched the standard JCPDS No. 09-0432. These results confirm the nucleation of hydroxyapatite (HA) on the surfaces of all coated specimens.

#### 3.2.3. Detection of the Surface Elastic Modulus of Each Group of Specimens

**Figure 10:**
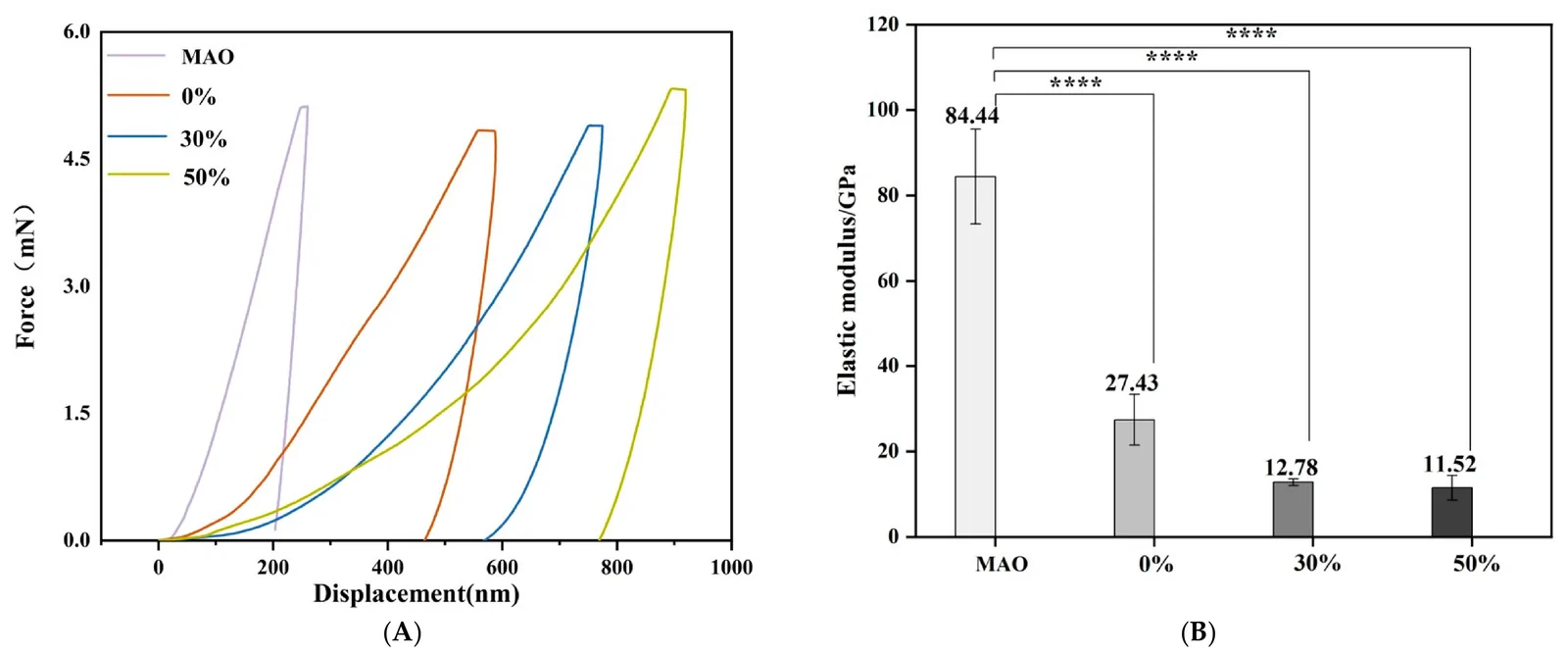
(**A**) Load–displacement curve of each group of specimens during the loading–unloading process, and (**B**) comparison diagram the of elastic modulus of each group of specimens; ****, *p* < 0.0001; *n* = 3.

Figure 10A presents nano-indentation force–displacement loading curves of four groups of specimens: MAO, 0%, 30% and 50%. The loading curves of the organic composite coating groups exhibit remarkable differences compared with those of the MAO group. All specimens reached a maximum load of 4.5–6.0 mN. With the increase in SPI content, the plastic deformation capacity of the coatings was enhanced, and the indentation displacement increased gradually under the same load. The sequence of indentation displacement was ranked as MAO < 0% < 30% < 50%.

Figure 10B displays the surface elastic modulus of each specimen measured by nano-indentation loading–unloading mechanical tests. The elastic modulus of the pure titanium substrate is approximately 110–120 GPa, which creates a prominent modulus mismatch with human cortical bone (10–40 GPa) and easily induces the stress shielding effect. The surface elastic modulus of the MAO group was 84.4 ± 11.1 GPa, and the elastic moduli of the 0%, 30% and 50% organic composite coatings were 27.4 ± 5.9 GPa, 12.8 ± 0.8 GPa and 11.5 ± 2.9 GPa, respectively. The surface elastic moduli of the MAO group and three SPI composite coating groups were significantly lower than that of the pure titanium substrate (*p* < 0.05). In addition, the surface elastic moduli of all organic coatings fell within the modulus range of human cortical bone, which effectively alleviates the mechanical discrepancy between implants and bone tissue and realizes excellent mechanical matching with host bone.

#### 3.2.4. Adhesion Test of Each Coating Specimen

A diamond stylus scratch tester was employed to scratch the coating surface. The critical load was defined as the applied load at which coating failure occurred, and this value was used to evaluate the interfacial adhesion strength between the coating and the titanium substrate [33].

**Figure 11:**
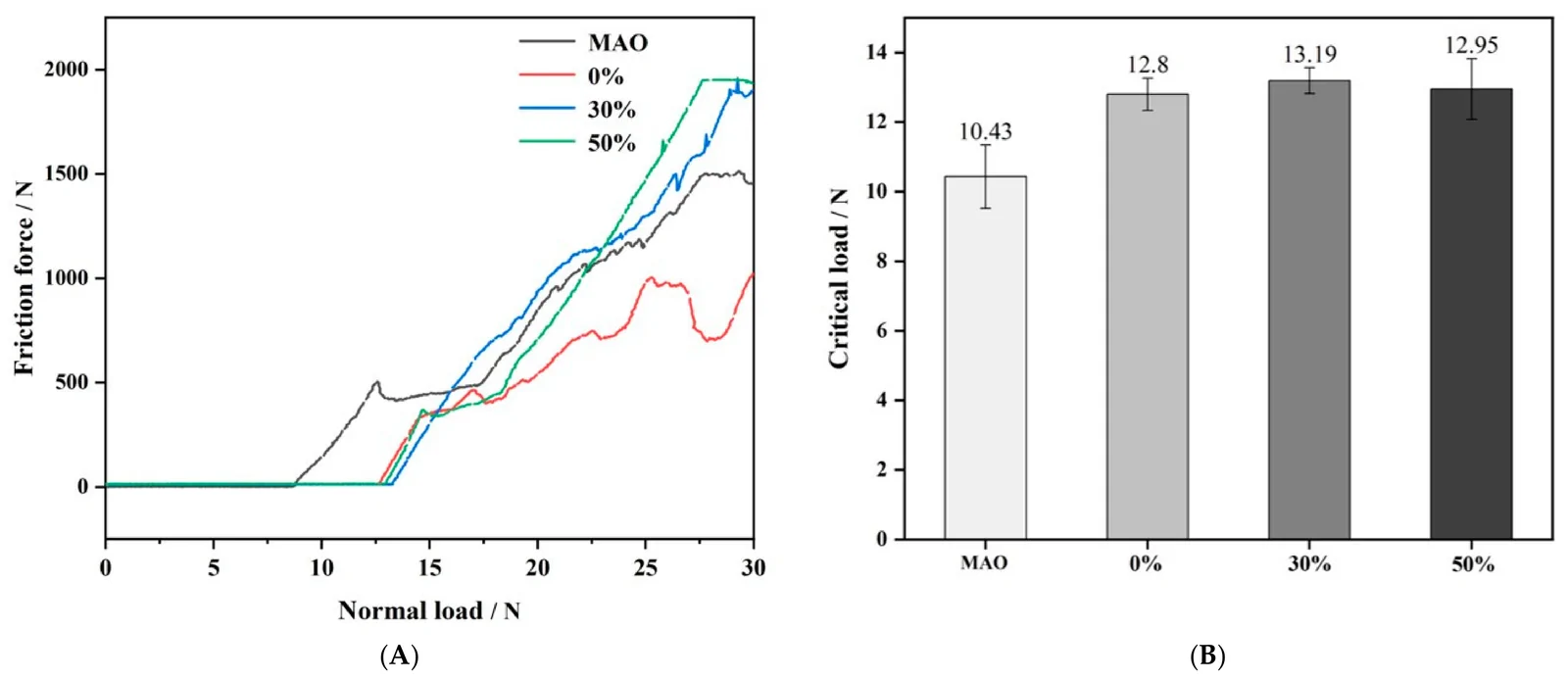
(**A**) Critical load of each group of specimens, and (**B**) contrast diagram of adhesion of each group of specimens.

As depicted in Figure 11A,B, the adhesion strengths of the 0%, 30% and 50% SPI groups were 12.80 ± 0.46 N, 13.19 ± 0.37 N and 12.94 ± 0.87 N, respectively. All of these values were significantly higher than that of the MAO group (10.43 ± 0.91 N), with statistically significant differences. The results reveal that SPI incorporation enhances the interfacial adhesion between the composite coating and the substrate. Nevertheless, the adhesion strength of the 50% SPI group was slightly lower than that of the 30% SPI group. This phenomenon suggests that once the coating thickness exceeds an optimal value, the adhesion strength no longer increases proportionally with coating thickness.

#### 3.2.5. Corrosion Resistance Test of Each Group of Coating Specimens

**Figure 12:**
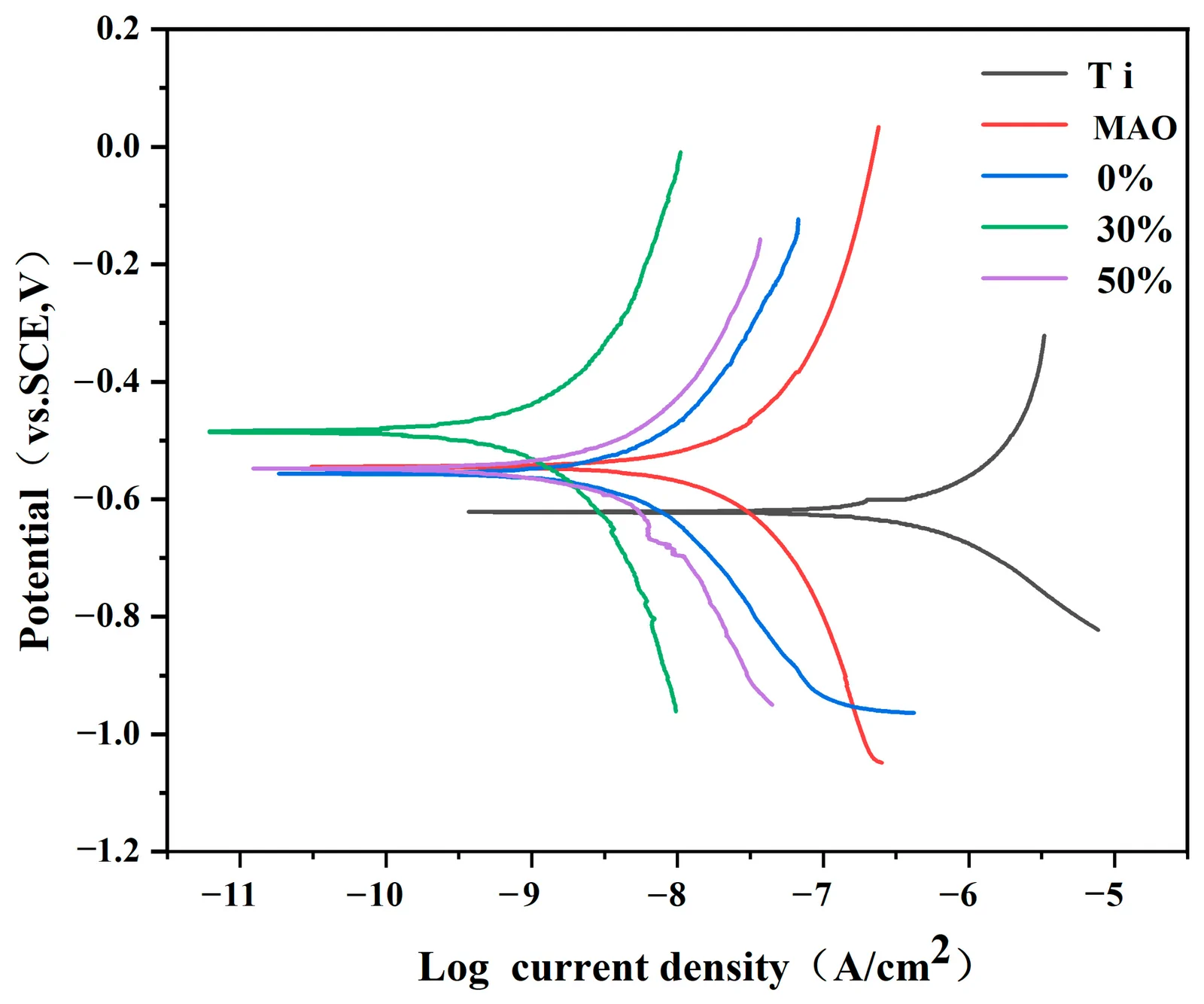
Potentiodynamic polarization curves of each group of specimens.

Potentiodynamic polarization testing is a classic electrochemical method for analyzing corrosion behavior and evaluating surface anticorrosion performance [34]. Figure 12 displays the potentiodynamic polarization curves of all experimental groups. Generally, a higher self-corrosion potential (E_corr_) indicates a lower corrosion tendency, while a lower corrosion current density (I_corr_) corresponds to a slower corrosion rate. The electrochemical parameters were obtained by Tafel slope fitting and are summarized in Table 3. The results showed that the MAO, 0%, 30%, and 50% groups exhibited higher E_corr_ values than the pure Ti group, suggesting an increased tendency for corrosion resistance. In addition, all coating groups presented lower corrosion current densities than pure titanium, indicating a significantly reduced corrosion rate.

### 3.3. Biological Performance Testing

#### 3.3.1. Hemolysis Test

**Figure 13:**
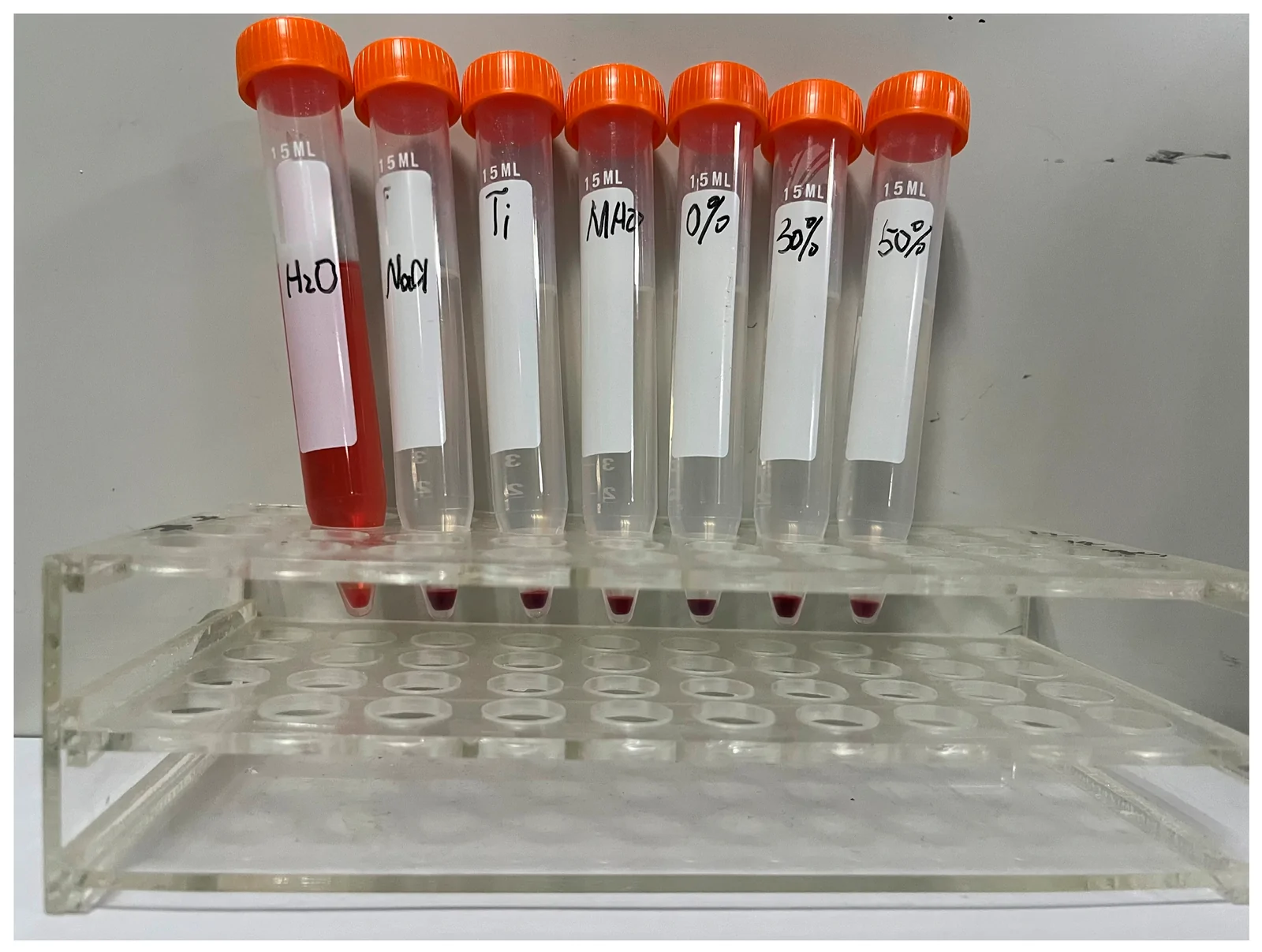
Appearance of the hemolysis rate of each group of specimens.

As shown in Figure 13, significant visual differences were observed in the centrifuged erythrocyte suspension among different groups. In the positive control group, no red blood cell precipitation was found at the bottom of the centrifuge tube, and the supernatant appeared bright red. This phenomenon indicated severe hemolysis, in which erythrocyte rupture led to the release of intracellular hemoglobin into the solution. In contrast, the supernatants of the negative control group and all five experimental groups remained clear and transparent, with obvious red blood cell sedimentation at the tube bottom. No evident hemolytic reaction was observed in any of the coating groups.

**Figure 14:**
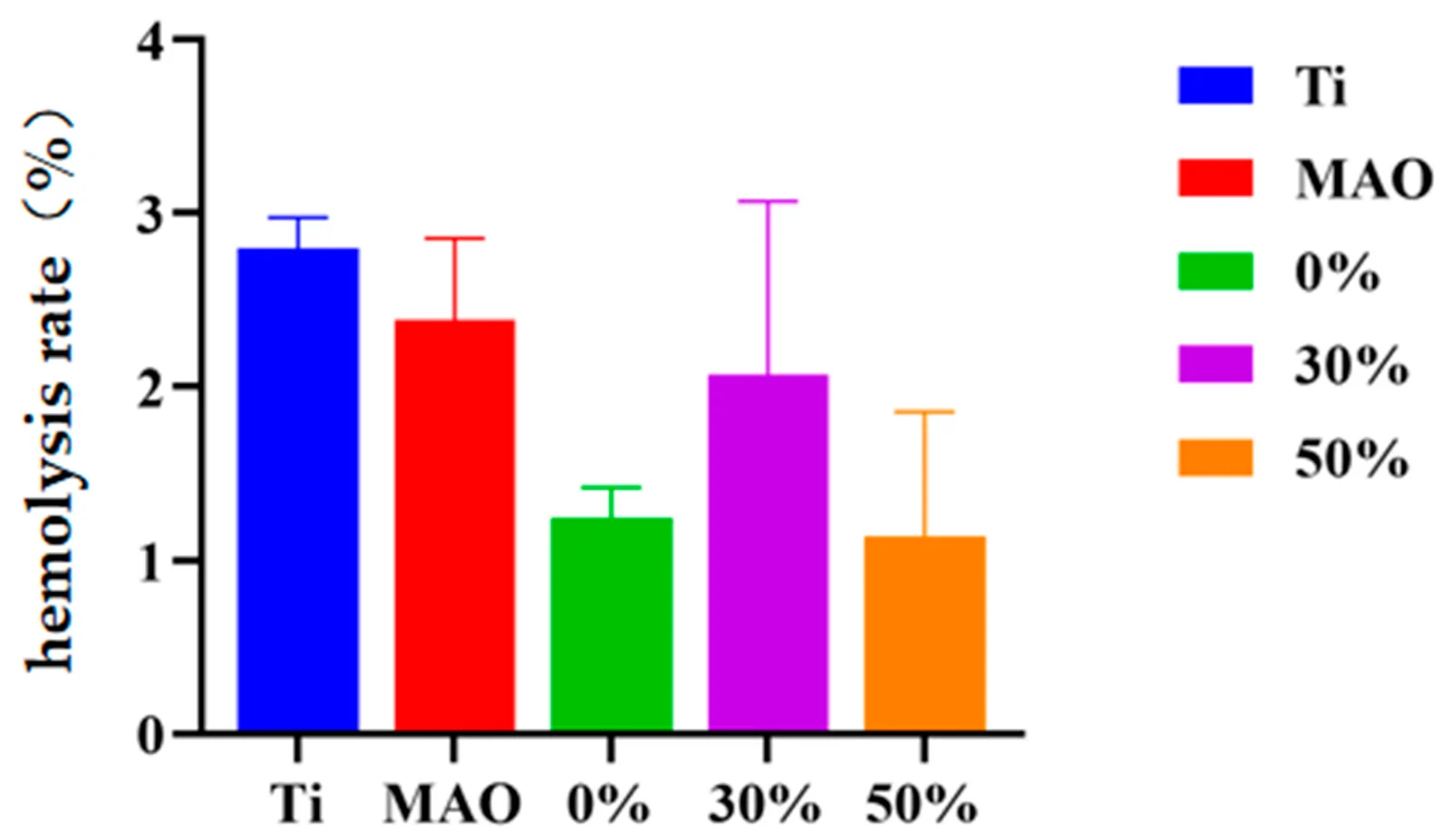
Hemolysis rate histogram.

Figure 14 presents the hemolysis rate of all specimens, which was below 3% for every group. This value meets the criteria specified in ISO 10993-4:2022, “Biological Evaluation of Medical Devices—Part 4: Selection of Tests for Interactions with Blood” [35], which stipulates an acceptable hemolysis rate below 5%. The results confirm that the pure Ti, MAO, 0% SPI, 30% SPI, and 50% SPI groups do not induce obvious hemolysis.

#### 3.3.2. Cell Experiment Results

##### Proliferation Results of CCK-8 Cells Co-Cultured with MC3T3-E1 in Each Group of Specimens

**Figure 15:**
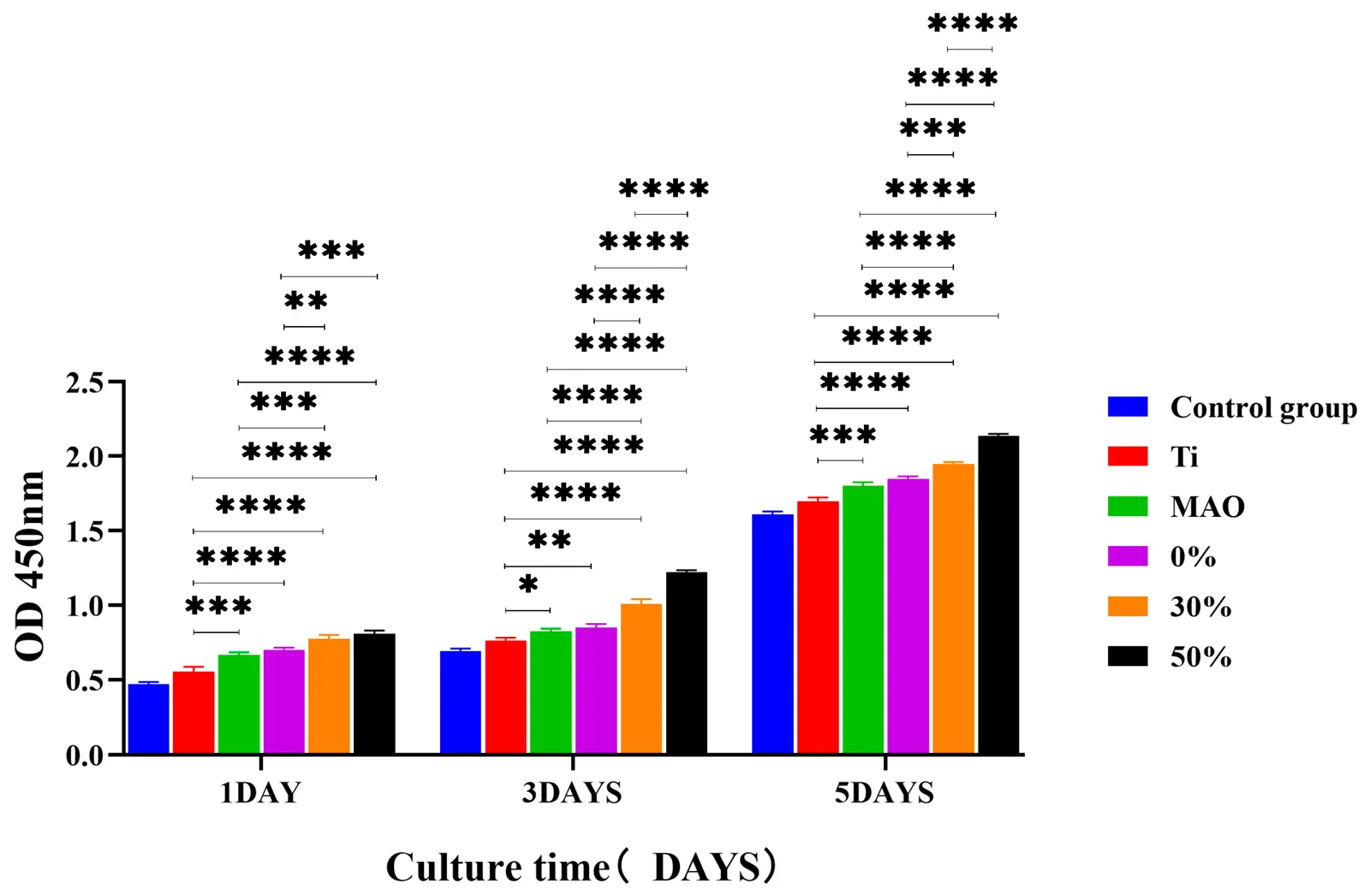
The effects of CCK-8 detection on the proliferation of MC3T3-E1 cells: *, *p* < 0.05; **, *p* < 0.01; ***, *p* < 0.001; ****, *p* < 0.0001; *n* = 3.

The proliferation of MC3T3-E1 cells co-cultured with different specimens was evaluated via the CCK-8 assay (Figure 15). All groups exhibited steady cell growth after incubation for 1, 3, and 5 days. Significant differences were observed between the five material groups and the blank control group at all time points, indicating that all specimens possessed favorable biocompatibility. Furthermore, the MAO, 0%, 30%, and 50% groups showed significantly higher cell proliferation than the pure Ti group at each time point. These results demonstrated that both MAO modification and organic coating treatment effectively improved the surface biocompatibility of pure titanium. In addition, the 30% and 50% SPI groups exhibited superior cell proliferation compared with the Ti, MAO, and 0% groups throughout the culture period. These findings suggest that the incorporation of SPI further promotes cell adhesion and growth, with the 50% SPI group showing the optimal proliferative effect.

**Figure 16:**
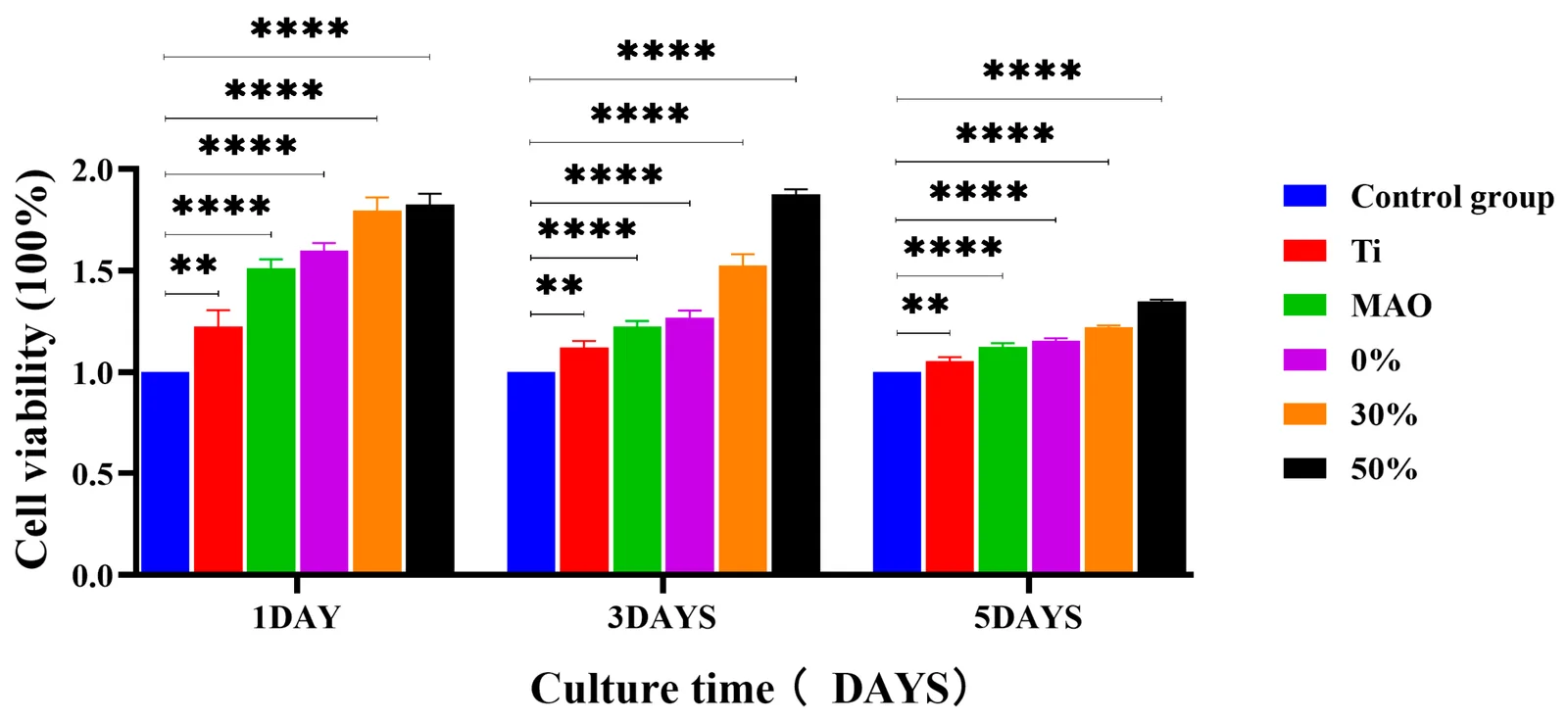
Effects of each group of specimens on the proliferation rate of MC3T3-E1 cells. **, *p* < 0.01; ****, *p* < 0.0001; *n* = 3.

Figure 16 illustrates the relative cell viability of MC3T3-E1 cells after co-culture with different specimens. At all detected time points, the five experimental groups exhibited significantly higher cell proliferation rates than the negative control group. These results confirm that all prepared coatings possess no cytotoxicity and effectively promote cell growth.

According to the ISO 10993-5 [36] evaluation criteria (Table 4), the cytotoxicity grade of all experimental groups was rated as Grade 0, demonstrating the excellent biocompatibility of the modified titanium surfaces.

##### Adhesion and Growth of Cells on Each Group of Specimens

**Figure 17:**
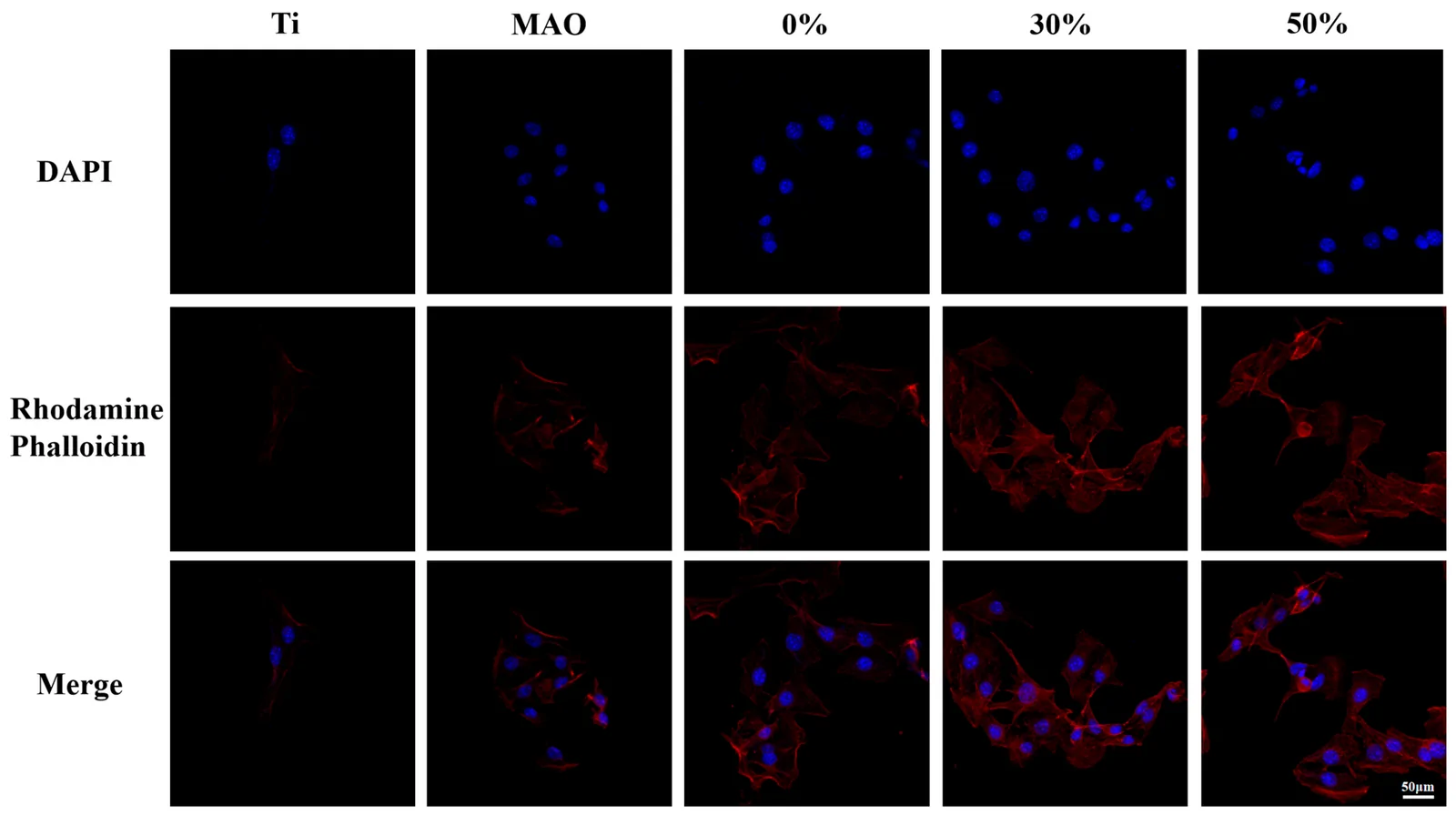
Laser confocal results of MC3T3-E1 cells co-cultured with each group of specimens for 48 h (Scalebar = 50 μm).

Laser confocal microscopy images of MC3T3-E1 cells after 48 h of co-culture are displayed in Figure 17. Cells adhered and spread on the surfaces of all groups, covering the specimen surfaces to varying degrees. In the pure Ti group, fewer and smaller cells were observed with a flat morphology, indicating relatively poor cell growth on the polished titanium surface. In comparison, cells in the MAO and 0% SPI groups presented spindle-shaped and polygonal morphologies with extended cytoskeletons, demonstrating improved cell spreading and active growth. Furthermore, cells seeded in the 30% and 50% SPI groups exhibited significantly larger spreading areas and more elongated cytoskeletons than those on the Ti, MAO, and 0% groups. Abundant filamentous pseudopodia were clearly visible, suggesting that the SPI-containing composite coatings provided the most favorable microenvironment for cell adhesion and growth.

**Figure 18:**
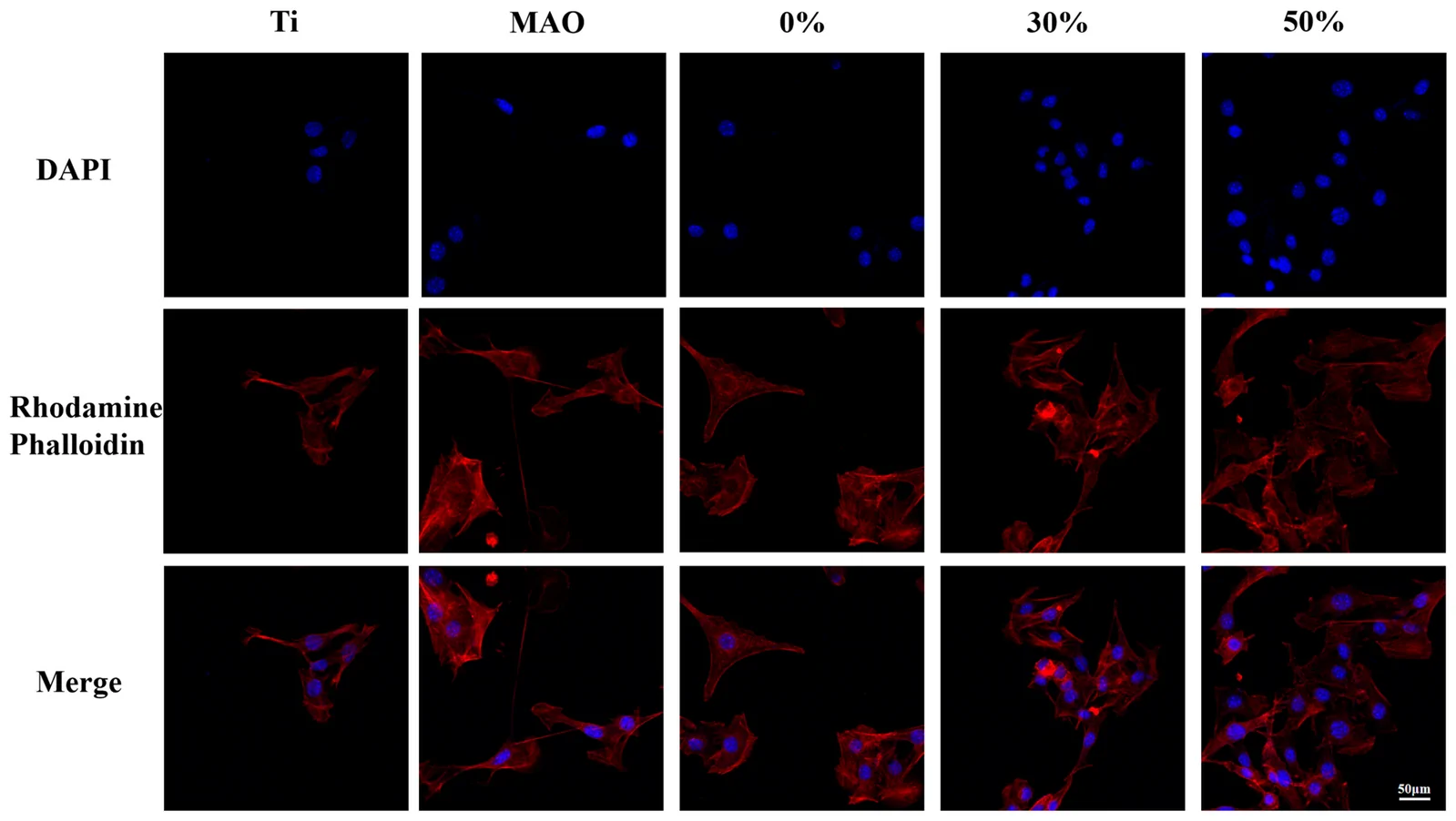
Laser confocal results of MC3T3-E1 cells co-cultured with each group of specimens for 72 h (Scalebar = 50 μm).

Figure 18 presents laser scanning confocal images of MC3T3-E1 cells cultured on various specimens for 72 h. Compared with the 48 h culture results, cells in all groups exhibited more extensive spreading after prolonged incubation. At 72 h, the 30% and 50% SPI groups displayed a greater number of cells and more pronounced cytoskeletal extension than the Ti, MAO, and 0% SPI groups. Filamentous pseudopodia interconnected to form continuous networks, and the cells showed an obvious three-dimensional morphology. These findings indicate that the introduction of SPI, along with increased SPI content, optimizes the surface biocompatibility of materials and greatly facilitates cell adhesion and spreading.

##### Alkaline Phosphatase (ALP) Activity

**Figure 19:**
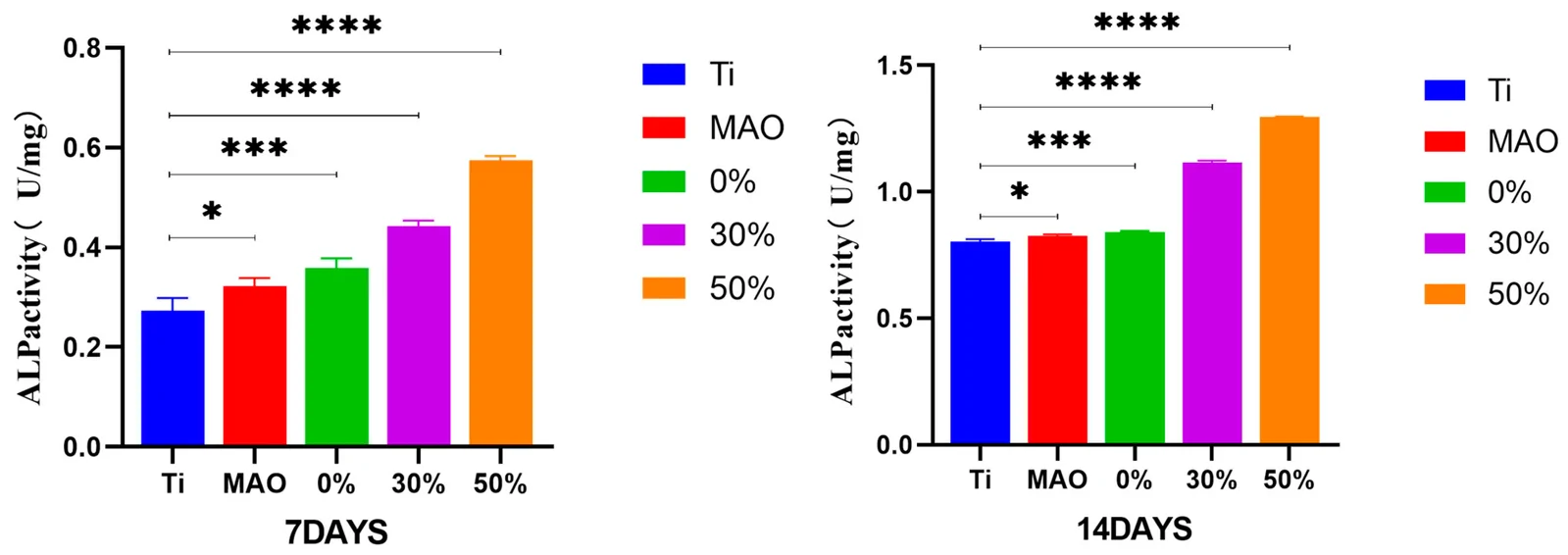
Effects of each group of specimens on ALP expression in MC3T3-E1 cells. *, *p* < 0.05; ***, *p* < 0.001; ****, *p* < 0.0001; *n* = 3.

Osteoblasts participate in multiple stages of osseointegration around titanium implants, including cell adhesion, proliferation, and osteogenic differentiation. As a crucial early biomarker of osteogenic differentiation, alkaline phosphatase (ALP) plays a vital role in osteoblast maturation and subsequent bone formation [37]. Figure 19 displays the ALP activity of MC3T3-E1 cells after 7 and 14 days of culture on different specimens. The osteogenic differentiation behavior of MC3T3-E1 cells can be effectively evaluated by detecting ALP expression levels at different culture stages. At both 7 and 14 days, the MAO, 0%, 30%, and 50% groups exhibited significantly higher ALP activity than the pure Ti group (*p* < 0.05). Overall, ALP expression in all groups increased with prolonged culture time. On day 7, the 30% and 50% SPI groups showed markedly higher ALP activity than the Ti, MAO, and 0% groups. On day 14, no significant difference in ALP activity was observed among the Ti, MAO, and 0% groups. Although the ALP activity of the 30% and 50% SPI groups slightly decreased from day 7 to day 14, their osteogenic activities remained significantly higher than those of the other three groups throughout the culture period.

##### Alizarin Red Staining

**Figure 20:**
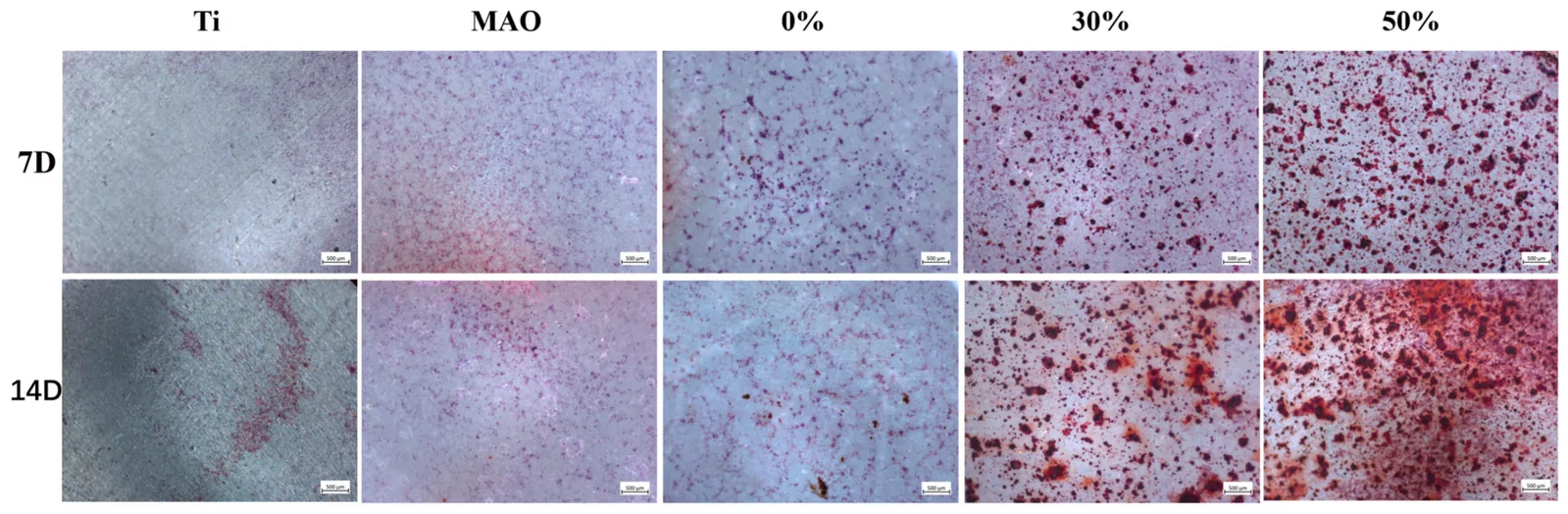
Alizarin red staining of samples from each group co-cultured with MC3T3-E1 cells in mineralization induction medium for 7 and 14 days.

Figure 20 shows stereomicroscopic images of alizarin red staining for MC3T3-E1 cells after 7 and 14 days of osteogenic induction. Orange-red stained deposits represent calcium nodules, which are characteristic markers of late-stage osteogenic differentiation [38]. Few visible mineralized deposits were observed in the pure Ti and MAO groups. In contrast, abundant calcium nodules were formed on the surfaces of 0%, 30% and 50% SPI/PLA–PCL composite coatings. Among the coating groups, mineralized nodules became increasingly numerous and larger with rising SPI content, and the staining intensity gradually strengthened, demonstrating enhanced osteogenic mineralization capacity. The mineralization effect at 14 days was markedly superior to that at 7 days.

**Figure 21:**
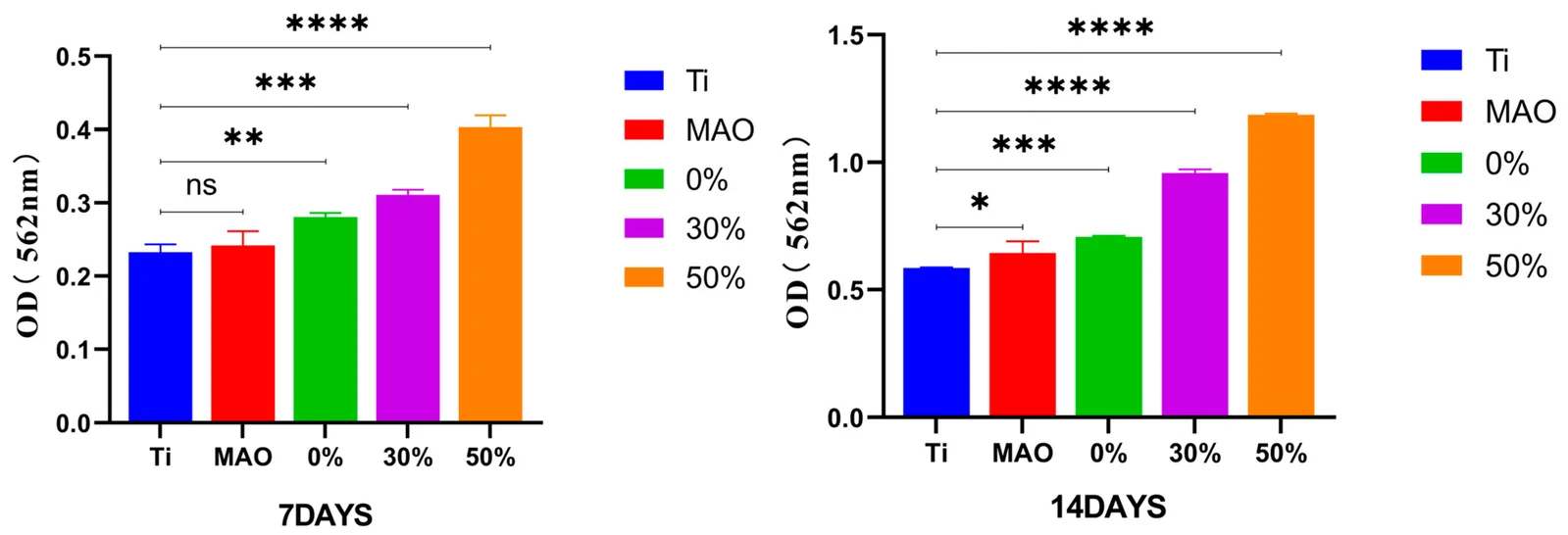
Semi-quantitative analysis results of alizarin red staining of samples and MC3T3-E1 cells co-cultured in mineralization induction medium for 7 and 14 days. *, *p* < 0.05; **, *p* < 0.01; ***, *p* < 0.001; ****, *p* < 0.0001; *n* = 3.

The semi-quantitative analysis results presented in Figure 21 reveal that the mineralization levels of the 30% and 50% SPI groups at days 7 and 14 were significantly higher than those of the Ti, MAO, and 0% groups at the corresponding time points. These results confirm that SPI effectively accelerates the osteogenic differentiation of MC3T3-E1 cells. Among all experimental groups, the 50% SPI group exhibited the highest degree of mineralization, suggesting its strongest capacity to induce osteogenic differentiation.

## 4. Discussion

### 4.1. Synthesis and Characterization of SPI/PLA-PCL Composite Coating on Pure Titanium

Porous MAO ceramic interlayers were constructed on pure titanium substrates to build a mechanical interlocking interface, solving the poor adhesion defect of direct organic coating deposition on smooth titanium surfaces. The SPI/PLA–PCL composite coatings were successfully fabricated on MAO-modified titanium via spin coating [30]. SEM and EDS results verified that the PLA–PCL matrix completely encapsulated the porous MAO structure, with sulfur elemental signals originating from SPI uniformly distributed on the coating surface and no substrate exposure, confirming the integrity and coverage of the composite film.

FT-IR and XRD analyses further demonstrated that SPI was uniformly dispersed in the PLA–PCL matrix through physical blending. No new chemical bonds or crystalline phases were generated during preparation, indicating that the composite system maintained stable physical compatibility without chemical-reaction-induced structural defects. The physical doping strategy endows the coating with a continuous and homogeneous microstructure, laying a structural foundation for the subsequent adjustable wettability, degradability, and biological performance of the composite coating.

### 4.2. Analysis of the Water Contact Angle of Each Group of Specimens

Surface hydrophilicity is a critical surface parameter that profoundly modulates initial cell adhesion, spreading, and subsequent osteogenic behaviors [33,39]. Generally, a contact angle below 90° indicates favorable surface wettability, which is beneficial for protein adsorption and cell colonization on implant surfaces [40,41,42,43].

The porous topography generated by MAO treatment effectively improved the hydrophilicity of the titanium substrate. In contrast, the intrinsic hydrophobic nature of PLA–PCL slightly increased the surface contact angle after organic coating deposition. Nevertheless, the incorporation of SPI significantly optimized the surface wettability of the composite coatings. Owing to the abundance of hydrophilic functional groups (–COOH, –OH, and –NH_2_) on SPI molecular chains, the hydrophobicity of the polyester matrix was effectively neutralized. Accordingly, the surface hydrophilicity was progressively enhanced with increasing SPI content.

The improved hydrophilic surface created by SPI modification provides a biologically favorable microenvironment for early cell attachment and proliferation, which helps accelerate the osseointegration process of titanium implants.

### 4.3. Roughness Analysis of Each Group of Specimens

Surface roughness serves as a crucial topographical factor that cooperatively regulates surface wettability and cell–material interactions [44]. In this study, MAO treatment introduced abundant microscale porous structures on titanium surfaces, which effectively increased surface roughness and specific surface area compared with polished pure titanium [45].

Further incorporation of SPI particles into the PLA–PCL matrix generated additional microscale surface protrusions, further enriching the surface microstructure and gradually increasing surface roughness with an elevated SPI proportion. Appropriate surface roughness provides more mechanical anchoring sites for osteoblast attachment and facilitates cell recognition and spreading. Consequently, the optimized multilevel topography induced by MAO and SPI modification significantly improves the surface bioactivity of titanium implants [46].

### 4.4. In Vitro Degradation Analysis of Each Group of Coating Specimens

Appropriate degradation kinetics are a fundamental requirement for bone regeneration coatings, which guarantee the gradual replacement of the implant material by newly formed bone tissue. The degradation behavior of the MAO layer and SPI/PLA-PCL composite coatings was evaluated via long-term immersion in standard PBS buffer.

The porous MAO ceramic layer possessed remarkable chemical inertness in a PBS environment. The porous architecture only allowed passive liquid infiltration at the initial stage, and the ceramic phase was resistant to hydrolytic erosion under physiological conditions, which endowed the MAO modified surface with outstanding dimensional stability during long-term immersion.

For PLA–PCL-based organic coatings, water infiltration is the prerequisite for polyester hydrolysis. The introduction of soybean protein isolate destroyed the tight packing of PLA–PCL molecular chains, reducing the compactness of the entire coating film. This structural change greatly facilitated the diffusion of PBS solution into the interior of the coating, shortened the induction period of the hydrolytic reaction, and accelerated the cleavage of ester linkages in the polymer backbone [47]. Moreover, SPI itself is a hydrophilic biomacromolecule that is prone to hydrolytic decomposition in aqueous media, and its rapid degradation can further promote the hydrolysis reaction of the surrounding polyester matrix, resulting in the enhancement of the overall degradation rate of the composite system with increasing SPI proportion [48].

PBS has a strong buffering capacity, which can counteract acidic small molecules produced during the hydrolysis of PLA–PCL. Even during the vigorous degradation stage of the composite coatings, the buffer system avoided severe acidification of the local microenvironment. All groups maintained a nearly neutral pH condition throughout the whole test cycle, which eliminated the risk of inflammatory response caused by excessive local acidity around the implant.

Most importantly, after the rapid degradation stage, the residual composite structure presented a moderate and sustained degradation rate. Such a degradation mode is highly consistent with the speed of bone defect repair, enabling the synchronous replacement of the degradable coating with newly formed bone tissue, which is conducive to the long-term stable osseointegration of implants.

### 4.5. In Vitro Mineralization Analysis of Each Group of Specimens

Biomineralization ability reflects the osteoinductive potential of implant coatings and is the key to forming stable bone–implant bonding [49,50]. SPI contains a large number of carboxyl and hydroxyl active groups, which can specifically chelate free Ca^2+^ in SBF solution and act as heterogeneous nucleation centers for hydroxyapatite crystallization [51]. With the increase in the SPI doping ratio, the number of surface mineral nucleation sites increases significantly, which greatly enhances the mineral deposition efficiency. In the long-term immersion process, the mild degradation of the composite coating continuously exposes internal active functional groups, providing sustainable nucleation sites for apatite growth and realizing continuous mineral deposition. XRD results confirmed the formation of crystalline hydroxyapatite on all SPI-modified coating surfaces, indicating that SPI functional modification successfully endows titanium implants with excellent in vitro osteoinductive mineralization ability.

### 4.6. Analysis of the Elastic Modulus of Each Group of Specimens

The excessive elastic modulus of pure titanium causes severe mechanical mismatch with cortical bone, resulting in a stress shielding effect, bone resorption and poor long-term osseointegration [52,53]. The MAO ceramic layer reduces the surface modulus of titanium by constructing a porous gradient structure. On this basis, the flexible biological macromolecule SPI further optimizes the mechanical properties of PLA–PCL matrix.

The incorporation of SPI softens the rigid polyester molecular network, reduces the surface hardness and elastic modulus of the coating, and adjusts the surface modulus of the composite coating to the optimal range matching human cortical bone. The optimized modulus matching eliminates the interfacial stress difference during load bearing, weakens the stress shielding effect, homogenizes the interfacial stress distribution of implants, and provides a favorable mechanical microenvironment for bone tissue remodeling and stable long-term osseointegration [54].

### 4.7. Analysis of Coating Adhesion in Each Group

The interfacial adhesion strength between the coating and titanium substrate was measured using an automatic scratch-testing system equipped with a diamond-shaped stylus. During the scratch-loading test, the normal load increased continuously, and the real-time friction–force–normal load curve was recorded (Figure 11A). The critical load is defined as the normal load corresponding to the abrupt inflection point on the friction–force curve, at which the coating begins to delaminate and fail from the substrate surface [55]. The magnitude of this critical-load value represents the interfacial-binding performance; a higher critical load indicates stronger coating–substrate adhesion.

Combined with the friction–load curves (Figure 11A) and quantitative critical-load histogram (Figure 11B), the critical loads of the 0% SPI, 30% SPI and 50% SPI groups are 12.80 ± 0.46 N, 13.19 ± 0.37 N and 12.94 ± 0.87 N, respectively. All these values are significantly higher than that of the MAO-only group (10.43 ± 0.91 N). Interfacial bonding strength determines the long-term structural stability of coatings under in vivo physiological loading. The porous architecture of the MAO layer provides mechanical interlocking anchoring sites for the overlying organic PLA–PCL-based film, which greatly improves the interfacial bonding force compared with untreated pure titanium substrates.

Moderate SPI doping (30% SPI group) optimizes the internal microstructure of the PLA–PCL matrix, eliminates microcracks and pore-induced defects inside the coating, improves the interfacial compatibility between the organic phase and MAO ceramic layer, and enhances interfacial interaction forces, thereby increasing the overall adhesion strength. The 30% SPI sample exhibits the highest critical load (13.19 ± 0.37 N) among all experimental groups. In contrast, excessive SPI addition (50% SPI) triggers macromolecular agglomeration and introduces abundant internal structural defects and discontinuous polymer networks. These defects break the overall integrity of the coating–substrate interface, and the friction-curve mutation load correspondingly declines (12.94 ± 0.87 N). Therefore, the 30% SPI group achieves optimal interfacial adhesion. This finding demonstrates that moderate modification with biological macromolecules facilitates the fabrication of structurally stable coating systems, whereas excessive SPI doping inevitably deteriorates the interfacial mechanical properties.

### 4.8. Corrosion Resistance Analysis of Each Group of Specimens

The electrochemical corrosion resistance of all coating groups was characterized via potentiodynamic polarization measurements, with corrosion potential (E_corr_) and corrosion current density (I_corr_) adopted as the key evaluation indices [56,57,58]. In general, a higher E_corr_ corresponds to a lower corrosion tendency, while a smaller I_corr_ indicates superior anti-corrosion performance [59].

As shown in Figure 12, all MAO-modified and composite coating groups displayed a positively shifted corrosion potential compared with the bare titanium substrate, confirming the effective enhancement of surface corrosion resistance. The oxide phases, amorphous structures, and high-potential precipitates formed within the MAO layer significantly suppress electron release and anodic dissolution of the titanium substrate, thus inhibiting the intrinsic electrochemical corrosion process [57].

The outer SPI/PLA–PCL composite coating acts as a dense physical barrier to isolate corrosive chloride ions from the substrate and restrain interfacial electrochemical corrosion reactions [34]. Among all experimental groups, the 30% SPI composite coating achieved the optimal anti-corrosion performance. Moderate and homogeneous incorporation of SPI optimized the microstructure, compactness and structural integrity of the coating, blocking the permeation and diffusion of chloride ions, as well as their interfacial adsorption, which provided long-term and reliable protection for the titanium substrate. In contrast, excessive SPI content increased the coating thickness and induced numerous internal pore defects, damaging the structural continuity of the polymeric film. These microstructural defects constructed fast diffusion channels for corrosive ions and accelerated interfacial corrosion, leading to a reduced corrosion potential, an increased corrosion current density and deteriorated overall corrosion resistance of the composite coating.

### 4.9. Blood Compatibility Evaluation

The hemolysis test is a basic experiment in the detection of acute hemolysis and biological safety of materials [60,61]. When the implant is implanted into the body, the physical and chemical properties of the material itself, or the decomposed and precipitated substances of the material, may cause the rupture of red blood cells, the release of hemoglobin inside the red blood cells, and a hemolytic reaction [62,63]. The composite organic coating on the surface of pure titanium designed in this project needs to be in direct contact with bone tissue and must also come into contact with blood cells in the bone marrow cavity. Therefore, the hemolysis test of this material is an important part of its biocompatibility.

In this experiment, the hemolysis test results are shown in Figure 14, which shows that the hemolysis rate of each coating sample group is less than 3%, which meets the relevant requirements of the standard hemolysis test (<5%) in ISO 10993-4:2022, “Biological Evaluation of Medical Devices Part 4: Selection of Interaction Tests with Blood”. The stable physical and chemical structure of SPI/PLA–PCL composite coating avoids hemoglobin release and erythrocyte rupture after blood contact. The degradation products of SPI and polyester are non-toxic amino acids and small molecular aliphatic compounds, which do not induce hemolytic reactions. The results confirm that the modified titanium coating possesses excellent blood compatibility and meets the basic biosafety requirements for in vivo implantation.

### 4.10. Biocompatibility Analysis of Each Group of Specimens

#### 4.10.1. Evaluation of Cell Activity In Vitro

In this experiment, we co-cultured MC3T3-E1 cells with each group of test specimens. As shown in Figure 15, the CCK-8 cell proliferation results showed that at each time point, the MC3T3-E1 cell proliferation activity of each experimental group was higher than that of the control group (pure DMEM medium), confirming that the five groups of specimens exhibited good biocompatibility with MC3T3-E1 cells. It was also found that the 30% and 50% SPI/PLA–PCL composite coating groups could significantly promote cell proliferation compared with the Ti group, and the 50% SPI/PLA–PCL composite coating group had the best effect on promoting cell proliferation.

The CCK-8 results verified that SPI modification effectively enhanced the proliferation of MC3T3-E1 osteoblasts, which can be explained by two synergistic mechanisms. On the one hand, the incorporation of SPI optimized the surface hydrophilicity and hierarchical topological structure of the coating, creating a favorable interfacial microenvironment for cell adhesion and spreading. On the other hand, the gradual degradation of SPI releases abundant amino acids into the culture medium, providing nutritional support for cell metabolism and growth. No cytotoxicity was detected for any group, which confirms the satisfactory biosafety of the SPI/PLA–PCL composite coatings.

#### 4.10.2. Observation of Cell Morphology

DAPI is a blue fluorescent dye that can penetrate the cell membrane and is often used for cell nucleus staining. Phalloidin is a cyclic heptapeptide toxin derived from the toadstool Amanita phalloides. It selectively binds to filamentous actin F-actin with high affinity (Kd = 20 nM), and dyes actin F-actin red to observe the cytoskeleton. After the staining is completed, it is observed with a fluorescence microscope. The staining results showed that, at the two time points of 48 h (Figure 17) and 72 h (Figure 18), the number of cells in the 30% and 50% SPI/PLA–PCL composite coating groups was larger, the cell shape was more stretched, and the filopodia were connected into sheets. Additionally, the structure is more three-dimensional, with the 50% SPI/PLA–PCL composite coating being the best. This is because the addition of SPI and the increase in content improve the roughness and hydrophilicity of the coating surface, which can promote the colonization and adhesion of MC3T3-E1 cells.

#### 4.10.3. ALP Activity Evaluation

ALP is an enzyme produced by cells in the process of osteogenic differentiation [64], which is an important sign of osteoblast differentiation. The synthesis period of the bone matrix begins to appear, and the mineralization period reaches its peak. In the process of osteogenesis, its expression activity increases with the increase in cell differentiation [65]. The results of ALP activity in each group are shown in Figure 19. The ALP expression in all five groups increased with the extension of culture time. On the 7th and 14th days, the ALP expression was as follow,: 50% group > 30% group > 0% > Mao group > Ti group, with significant statistical differences. The ALP expression of 30% and 50%SPI/PLA-PCL composite coatings was significantly higher than that of the other three groups, and there was no significant difference between Ti and MAO groups. The ALP expression of the 50% SPI/PLA–PCL composite coating was the strongest at all time points, which may be because the adhesion and proliferation effect of the 50% SPI/PLA–PCL composite coating on MC3T3-E1 cells was the most significant. Compared with the other three groups with poor adhesion effects, a large number of MC3T3-E1 cells adhered to the surface of the 50% SPI/PLA–PCL composite coating, and it was more conducive to the differentiation of MC3T3-E1 cells. Under the action of osteogenic induction medium, MC3T3-E1 cells were more conducive to the directional differentiation of osteoblasts, and the ALP expression activity increased.

#### 4.10.4. Alizarin Red Dyeing and Semi-Quantitative Evaluation Analysis

Alizarin Red S staining is a classic assay for evaluating the osteogenic differentiation of osteoblasts. It can specifically chelate calcium ions in mineralized nodules to form stable orange-red calcium deposits, which serve as a critical biomarker for late-stage osteogenic maturation [66]. The staining observations and semi-quantitative results further verified the osteoinductive effect of SPI. Abundant polar functional groups on SPI molecules continuously capture calcium and phosphate ions in the culture medium to facilitate the generation and maturation of mineralized nodules. Meanwhile, gradual degradation of the composite coating constantly exposes fresh active sites to provide sufficient nucleation sites for mineral deposition. The 50% SPI group exhibited the most desirable mineralization performance, demonstrating that SPI modification significantly improves the late osteogenic differentiation capacity of the coating and endows titanium implants with favorable osteoinductivity for reliable osseointegration.

## 5. Conclusions

SPI/PLA-PCL composite coatings were successfully fabricated on pure titanium substrates. The 50% SPI/PLA–PCL coating exhibits optimal comprehensive physicochemical and biological performance, regulates the surface elastic modulus to match human cortical bone, and effectively improves surface hydrophilicity, roughness, corrosion resistance, biocompatibility and osteogenic capacity, showing great potential for the surface modification of titanium implants.

## Figures and Tables

**Table 1:** Main reagents.

Raw Material	Molecular Formula/Model	Manufacturer
Polylactic acid	(C_3_H_4_O_2_)n	Shanghai Macklin Biochemical Technology Co., Ltd., Shanghai, China
Polycaprolactone	(C_6_H_10_O_2_)n	Shanghai Macklin Biochemical Technology Co., Ltd.
Soy protein isolate	C_13_H_10_N_2_	Shanghai Macklin Biochemical Technology Co., Ltd.
Ammonium bicarbonate	NH_4_HCO_3_	Sinopharm Chemical Reagents Co., Ltd., Beijing, China
Methylene chloride	CH_2_Cl_2_	Shanghai Aladdin Biochemical Technology Co., Ltd., Shanghai, China
Polyvinyl alcohol	(C_2_H_4_O)n	Sinopharm Chemical Reagents Co., Ltd.
Triple-distilled water	H_2_O	Provided by the Life Science Experiment Center of Jiamusi University, Jiamusi, China
DMEM medium	C0891-500 mL	Shanghai Beyotime Biotechnology Co., Ltd., Shanghai, China
Fetal bovine serum	04-001-1A	BI (Biolnd), Kibbutz Beit Haemek, Israel
Penicillin–streptomycin antibodies	C0222	Shanghai Beyotime Biotechnology Co., Ltd.
0.25% trypsin digest	C0201-100 mL	Shanghai Beyotime Biotechnology Co., Ltd.
Alizarin red kit	C0140-100 mL	Shanghai Beyotime Biotechnology Co., Ltd.
Alkaline phosphatase kit	P0321M	Shanghai Beyotime Biotechnology Co., Ltd.
CCK8 kit		Aperbio Biotech (Suzhou) Technology Co., Ltd., Suzhou, China
MC3T3-E1 cells		Provided by the team of Professor Zhang Huiming of Basic Medicine of Jiamusi University, Jiamusi, China
PBS buffer	BL601A	Biosharp, Hefei, China
Simulated body fluids	G0390-500 mL	Solarbios Science & Technology Co., Ltd., Beijing, China
4% paraformaldehyde fixative	P0099-500 mL	Shanghai Beyotime Biotechnology Co., Ltd.
DAPI	BL105A	Biosharp Life Sciences, Hefei, China
Pencil peptide	BL1190A	Biosharp Life Sciences, Hefei, China

**Table 2:** Instruments and equipment.

Name	Model	Manufacturer
Electronic balance	FA2004	Shanghai Shunyu Hengping Scientific Instrument Co., Ltd., Shanghai, China
Magnetic stirrer	DF-101S	Gongyi Yuhua Instrument Co., Ltd., Gongyi, China
Ultrasonic cleaning machine	KQ-300DE	Kunshan Ultrasound Instrument Co., Ltd., Kunshan, China
Glue homogenizer	TC-218	No manufacturer information supplemented/Homogenizer, TC-218
Vacuum drying oven	DZK-6012	Shanghai Yiheng Scientific Instrument Co., Ltd., Shanghai, China
Scanning electron microscope	TESCAN MIRA LMS	TESCAN Brno, s.r.o., Brno, Czech Republic
Fourier transform infrared spectrometer	Thermo Scientific Nicolet iS20	Thermo Fisher Scientific, Waltham, MA, USA
Centrifuge	TG16G	Henan Beihong Industrial Co., Ltd., Zhengzhou, China
Freeze dryer	Vir Tis Wizard 2.0	SP Scientific, Warminster, PA 18974, USA
X-ray diffractometer.	BRUKER D8 ADVANCE	Bruker Corporation, Karlsruhe, Germany.
Atomic force microscopy	Bruker Icon	Bruker Corporation, Germany
Stereo microscope	Stemi 508	Carl Zeiss AG, Oberkochen, Germany
Laser scanning confocal microscopy	LSCM	Olympus, Tokyo, Japan
Constant-temperature water bath	HH-US-B	Jincheng Chunlan Experimental Instrument Factory in Jintan District, Changzhou, China
Fully automatic multifunctional microplate reader	BioTek Synergy HT	BioTek Instruments, Inc., Winooski, VT, USA
Automatic coating adhesion scratch tester	WS-2005	Shenzhen Time Peak Technology Co., Ltd., Shenzhen, China.
Nano-indenter model	PB1000.	Micro Nano (Hong Kong) Technology Co., Ltd., Hong Kong, China
Electric constant-temperature incubator	DHP-420	Changzhou Zhongjie Experimental Instrument Manufacturing Co., Ltd., Changzhou, China
Contact angle measuring instrument	JYC	Zhongchen Digital Technology Equipment Co., Ltd., Shanghai, China
Micro-arc oxidation equipment	WHD-20	Good Will Instrument Co., Ltd., New Taipei City, Taiwan

**Table 3:** Fitting data of polarization curve; self-corrosion potential and corrosion current density.

Samples	I_corr_ (A/cm^2^)	E_corr_ (V vs. Ag/AgCl)
**Ti**	4.19 × 10^−7^ ± 1.26 × 10^−6^	−0.73 ± 0.02
**MAO**	1.32 × 10^−6^ ± 3.97 × 10^−6^	−0.77 ± 0.12
**0%**	8.30 × 10^−8^ ± 1.22 × 10^−7^	−0.84 ± 0.08
**30%**	8.35 × 10^−9^ ± 6.45 × 10^−9^	−0.92 ± 0.06
**50%**	8.98 × 10^−7^ ± 8.94 × 10^−7^	−0.72 ± 0.06

**Table 4:** Corresponding relationship between the cell proliferation rate and grade of cytotoxic reaction.

Grade	Relative Proliferation Rate
0	≥100
1	80–99
2	50–79
3	30–49
4	0–29

## Data Availability

The original contributions presented in this study are included in the article. Further inquiries can be directed to the corresponding author.

## Abbreviations

The following abbreviations are used in this manuscript:

EDS	Energy-dispersive spectrometer
XRD	X-ray diffraction
SEM	Scanning electron microscope
FTIR	Fourier transform infrared spectroscopy
SPI	Soy protein isolate
PLA	Polylactide
PCL	Polycaprolactone
ALP	Alkaline phosphatase
ECM	Extracellular matrix
FBS	Fetal bovine serum
SBF	Simulated body fluid
PBS	Phosphate-buffered saline
OD	Optical density
